# Can Scientific Evidence Support Using Bangladeshi Traditional Medicinal Plants in the Treatment of Diarrhoea? A Review on Seven Plants

**DOI:** 10.3390/nu5051757

**Published:** 2013-05-22

**Authors:** Helle Wangensteen, Line Klarpås, Mahiuddin Alamgir, Anne B. C. Samuelsen, Karl E. Malterud

**Affiliations:** 1 Department of Pharmaceutical Chemistry-Pharmacognosy, School of Pharmacy, University of Oslo, P.O. Box 1068, Blindern, N-0316 Oslo, Norway; E-Mails: klarpasline@gmail.com (L.K.); a.b.c.samuelsen@farmasi.uio.no (A.B.C.S.); k.e.malterud@farmasi.uio.no (K.E.M.); 2 School of Chemistry, University of New South Wales, Sydney, NSW 2052, Australia; E-Mail: m19alamgir@yahoo.com

**Keywords:** diarrhoea, Bangladesh, traditional medicine, mangrove plants, *Diospyros peregrina*, *Heritiera littoralis*, *Ixora coccinea*, *Pongamia pinnata*, *Rhizophora mucronata*, *Xylocarpus granatum*, *Xylocarpus moluccensis*

## Abstract

Diarrhoea is a common disease which causes pain and may be deadly, especially in developing countries. In Bangladesh, diarrhoeal diseases affect thousands of people every year, and children are especially vulnerable. Bacterial toxins or viral infections are the most common cause of the disease. The diarrhoea outbreaks are often associated with flood affected areas with contaminated drinking water and an increased risk of spreading the water-borne disease. Not surprisingly, plants found in the near surroundings have been taken into use by the local community as medicine to treat diarrhoeal symptoms. These plants are cheaper and more easily available than conventional medicine. Our question is: What is the level of documentation supporting the use of these plants against diarrhoea and is their consumption safe? Do any of these plants have potential for further exploration? In this review, we have choosen seven plant species that are used in the treatment of diarrhoea; *Diospyros peregrina*, *Heritiera littoralis*, *Ixora coccinea*, *Pongamia pinnata*, *Rhizophora mucronata*, *Xylocarpus granatum*, and *Xylocarpus moluccensis*. Appearance and geographical distribution, traditional uses, chemical composition, and biological studies related to antidiarrhoeal activity will be presented. This review reveals that there is limited scientific evidence supporting the traditional use of these plants. Most promising are the barks from *D. peregrina*, *X. granatum* and *X. moluccensis* which contain tannins and have shown promising results in antidiarrhoeal mice models. The leaves of *P. pinnata* also show potential. We suggest these plants should be exploited further as possible traditional herbal remedies against diarrhoea including studies on efficacy, optimal dosage and safety.

## 1. Background

Diarrhoea is a common and serious disease in almost all tropical countries of the world. Particularly children are exposed to diarrhoea, and diarrhoea is the principal cause of morbidity and mortality among children in the developing world [1]. It is proposed that about 17% of all deaths in children up to 5 years are caused by diarrhoea, corresponding to 1.8 million deaths annually (estimates for 2000–2003). The countries in South-East Asia contribute significantly to this [2]. There has been a decline in mortality caused by diarrhoea in the last decades, but the morbidity remains high [1]. The need to provide clean drinking water and hygiene facilities still remains a huge challenge in developing countries today. Currently, 1.1 billion people lack access to safe water [3].

The World Health Organization has defined diarrhoea as the passage of three or more loose or liquid stools per day, or more frequently than normal for the individual. Disturbances in the transport of electrolytes and water in the intestines give rise to diarrhoea. There are four major mechanisms responsible for the pathophysiology in electrolyte and water transport: (1) increased luminal osmolarity; (2) increased electrolyte secretion; (3) decreased electrolyte absorption; and (4) deranged intestinal motility causing a decreased transit time [4]. Diarrhoea is usually a result of gastrointestinal infection, which can be caused by a variety of bacterial, viral and parasitic organisms. Infection is spread through contaminated food or drinking water, or from person to person as a result of poor personal hygiene. *Vibrio cholerae* together with enterotoxigenic *Escherichia coli*, enteropathogenic *E. coli*, *Shigella* spp., *Campylobacter* *jejuni*, and rotavirus are the most likely causes of diarrhoea in Bangladesh [5,6]. Infections with multiple pathogens are common, making the identification of the causative agent difficult. It may also be that multiple pathogens act synergistically to produce diarrhoeal symptoms [5]. There are differences in the causes of diarrhoea between patients of different ages. Enterotoxigenic *E. coli* was shown to be the commonest cause of diarrhoea in patients below two years of age, while in older children and adults, cholera was the most common cause of diarrhoea [6,7]. 

In order to study antidiarrhoeal effects appropriate models are necessary. The most common method is to study the inhibitory effect of a test compound in an animal model after induction of diarrhoea. The inducing agent may be castor oil, magnesium sulphate, prostaglandin E_2_ or arachidonic acid. These agents appear to induce diarrhoea by different mechanisms, e.g., castor oil increases peristaltic activity and alters the permeability of the intestinal mucosa to water and electrolytes, while magnesium sulphate is an osmotically active agent [8]. The gastrointestinal transit is often determined by measuring the transit of a charcoal plug or suspension and the distance traveled by the agent is expressed as a percentage of the total length of the intestine. In addition to the effect of the test compounds on the gastrointestinal system, it is of interest to investigate the effect on microorganisms causing diarrhoea, as well.

Herbal remedies continue to be the only therapeutic possibility for the majority of the global population. A number of traditional medicinal plants and their constituents from all over the world are reported as agents used to treat diarrhoea. Dragon’s blood (sap of *Croton lechleri*) is an example of a traditional antidiarrhoeal plant utilized in the production of the commercial Dragon’s blood extract named SP-303™ by Shaman Pharmaceuticals, Inc. [9]. Traditional cocoa preparations have been used by indigenous people of Central America to treat childhood diarrhoea and other intestinal ailments, as well [10]. Numerous spices and medicinal plants such as ginger, rhubarb, Galla Chinensis, cardamom, *Moringa oleifera*, *Anthocephalus cadamba* as well as green and black tea have also been used against diarrhoea, and have been shown to give certain effects in antidiarrhoeal studies [11,12,13,14,15,16,17]. The plant extracts can show antispasmodic effects, delay gastrointestinal transit, suppress gut motility, stimulate water adsorption or reduce electrolyte secretion [8]. Plant extracts may have an inhibitory effect on the microorganisms involved in the pathogenesis of diarrhoea, as well. In complex natural products, synergistic effects may contribute to effective antidiarrhoeal treatment. An herbal preparation containing *Myrtus communi*, *Aegle marmelos*, *Punica granatum*, *Phyllanthus emblica* and *Berberis vulgaris* was recently reported to be superior to the allopathic drug Furoxone (furazolidone) in a clinical study of diarrhoea treatment [18]. Proanthocyanidins are major constituents of *Croton lechleri* sap [19]. This class of compounds is common in plants used to treat diarrhoea [20], and it has been suggested that the proanthocyanidin-rich SP303™ may act by inhibition of fluid accumulation and chloride secretion [21].

The inhabitants of Bangladesh are highly affected by diarrhoeal diseases. One important reason is the annual heavy monsoon rainfall which makes disastrous floods followed by contamination of drinking water, thereby leading to spreading of water-borne diseases such as cholera. Cyclones and tsunamis are risk factors for cholera outbreaks, as well. The number of people affected by flooding is projected to increase as a result of the raising global average temperature, thus leading to an increased risk of water-borne diseases, e.g., diarrhoea. The risk for diarrhoeal mortality and disease is projected to increase by a factor of 1.09 until 2030 due to the climate changes [22]. The future need of effective antidiarrhoeal medications is therefore highly warranted. The plants presented in this review are growing in the Sundarbans mangrove forest. The Sundarbans is the largest single tract of mangrove ecosystem in the world, covering about 6000 km^2^ of Bangladesh and India [23]. There is a worry, however, that this mangrove forest may be dwindling [24,25]. Mangroves are usually found only in tropical climates, as consistently warm conditions are necessary for development and survival of this type of forest. These wetland ecosystems are among the most productive and diverse in the world, and a wide variety of biologically active natural products have been reported from mangrove forests [26]. The mangroves play a vital role for the local people [27]. It has been estimated that ca. four million people depend on the mangroves for their livelihood [23]. Fishery, seafood and honey are important sources of income; the mangroves also provide raw material for paper, wood and furniture industry. In addition, there might be a potential for local sales of herbal remedies as an income source. Bangladesh is in particular affected by tropical cyclones due to its geographical position, but mangroves play an important role reducing the impact of the cyclones and accompanying surges [23].

The aim of this review is to summarize the present knowledge of some traditional medicinal plants used against diarrhoea in the Sundarbans mangrove forest in Bangladesh and to consider whether this documentation support the use and safety of these plants. The plants discussed are *Diospyros peregrina*, *Heritiera littoralis*, *Ixora coccinea*, *Pongamia pinnata*, *Rhizophora mucronata*, *Xylocarpus granatum*, and *Xylocarpus moluccensis* (Table 1). These plants all grow in the Sundarbans. However, the scientific evidence for their antidiarrhoeal effects turns out to be limited for most of the plants. When we describe each plant, various traditional medicinal usages are given (not only uses against diarrhoeal related diseases). The chemical composition of each plant part is described in detail. Concerning the biological activities, we have primarily focused on studies related to antidiarrhoeal effects or studies dealing with effects on diarrhoea inducing microorganisms. When such studies have been conducted, the potential toxicity of each plant is described. Potential associations between observed biological activity and chemical composition in relation to antidiarrhoeal effects are briefly discussed. The literature sources used in this review are the SciFinder and PubMed databases and Google searches in the “grey” literature, as well as handbooks, reference works and articles from the archives of the authors.

**Table 1:** Traditional antidiarrhoeal plants from Sundarbans mangrove forest.

Scientific name	Synonym	English name	Family
*Diospyros peregrina* Gürke	*D. biflora* Blanco	Gaub Persimmon Riber Ebony	Ebenaceae
	*D. citrifolia* Wall. ex A.DC.		
	*D. embryopteris* Pers.		
	*D. glutinifera* (Roxb.) Wall.		
	*D. glutinosa* J.König ex Roxb.		
	*D. malabarica* (Desr.) Kostel.		
	*D. siamensis* Hochr.		
	*Embryopteris gelatinifera* G.Don		
	*E. glutinifera* Roxb.		
	*E. glutinifolia* Link		
	*E. peregrina* Gaertn.		
*Heritiera littoralis* Dryand.	*Amygdalus littoralis* (Dryand.) Kuntze	Looking-glass mangrove	Sterculiaceae
	*Balanopteris tothila* Gaertn.		
	*H. minor* Bojer		
*Ixora coccinea* L.	*Pavetta coccinea* (L.) Blume	Jungleflame ixora	Rubiaceae
		Scarlet jungleflame	
*Pongamia pinnata* (L.) Pierre	*Cytisus pinnatus* L. **	Indian beech tree Pongam tree	Fabaceae
	*Dalbergia arborea* Willd.		
	*Derris indica* (Lam.) Bennet		
	*Galedupa indica* Lam.		
	*G. pinnata* (L.) Taub.		
	*G. pungum* J.G.Gmel.		
	*Millettia pinnata* L.		
	*M. novo-guineensis* Kaneh. & Hatus.		
	*P. glabra* Vent.		
	*P. mitis* (L.) Kurz		
	*P. xerocarpa* Hassk.		
	*Pterocarpus flavus* Lour.		
	*Robinia mitis* L.		
*Rhizophora mucronata* Lam.	*Mangium candelarium* Rumphius **	True mangrove	Rhizophoraceae
	*R. candelaria* Wight & Am.		
	*R. longissima* Blanco		
	*R. macrorrhiza* Griff.		
*Xylocarpus granatum* König	*X. obovatus* A. Juss. **	Puzzle nut tree	Meliaceae
	*Carapa granatum* (Koen.) Alston	Cannon ball tree	
*Xylocarpus moluccensis* M. Roem.	*Carapa moluccensis* Lam.		Meliaceae

## 2. Traditional Antidiarrhoeal Plants from Bangladesh

### 2.1. *Diospyros peregrina* Gürke (Ebenaceae)

**Figure 1:**
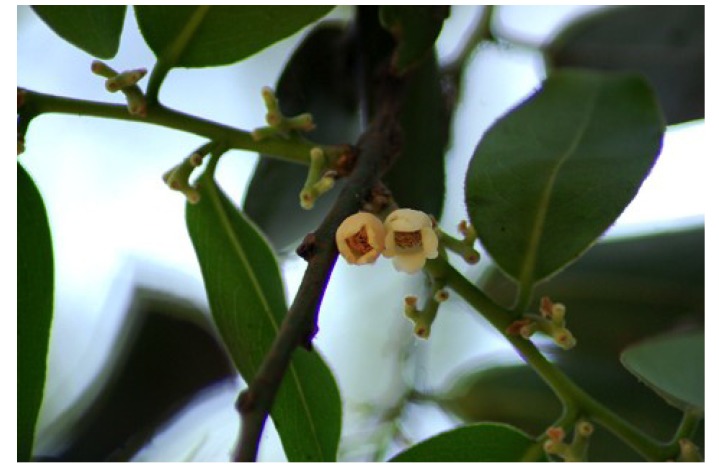
*Diospyros peregrina* (syn. *D. malabarica*) [29].

*Diospyros peregrina* (Figure 1) is a medium-sized evergreen tree up to 15 m high. It has bell-shaped flowers, the fruits are yellow when ripe, round and 4–8 cm in diameter [28]. The tree is indigenous to Bangladesh and India, and is also found in many other countries of Asia and America. The Bengali name is “gab”.

#### 2.1.1. Traditional Use and Plant Parts Used

The bark is astringent and has traditionally been used against dysentery and intermittent fevers. The seeds and the oil from the seeds are given as an astringent agent against diarrhoea. The ripe fruit has been used against biliousness, diseases of the blood, urinary losses, and stones in the urinary tract. The infusion of fruits is used as gargle in mouth ulcers and sore throats. The juice of the unripe fruit is used on wounds and ulcers, it has astringent properties, and it has also been used for the treatment of diabetes. The flowers are believed to be aphrodisiac and good for lumbago. They are also used in biliousness and diseases of the blood. The flowers and fruits are given to children with hiccough [28,30,31]. Tannins from *D. peregrina* are used for dyeing and in the tanning industry [32]. In Namibia, *D. peregrina* (unspecified plant parts) is employed against malaria [33]. Interestingly, an antiplasmodial activity of a stem bark extract of *D. peregrina* has been reported [34]. 

An extract of unripe fruits of the related species *D. melanoxylon* in milk has been reported to be used against diarrhoea in Madhya Pradesh, India [35].

#### 2.1.2. Chemical Composition (Figure 2)

*Bark*:

Triterpenoids: lupeol, betulin, betulinic acid, oleanolic acid [36];
Sterol: β-sitosterol [36];
Long-chain alcohol: myricyl alcohol [36];
Tannins: [30].

*Heartwood*:

Triterpenoid: Lupeol [37].

*Stems*:

Sterol: β-sitosterol [38];
Flavonoid: leucopelargonidin-3-*O*-α-l-rhamnopyranoside [38];
Aliphatic ketone: nonadecan-7-ol-2-one [39].

*Leaves*:

Triterpenoids: betulin, oleanolic acid, peregrinol [40,41];
Sterol: β-sitosterol [40,41].

*Fruits*:

Triterpenoids: peregrinol, lupeol, betulin, betulinic acid, taraxerone, marsformosanone [32,42,43];
Sterols: β-sitosterol, β-sitosterol-d-glucoside [32];
Flavonoids: furano-(2″,3″,7,8)-3′,5′-dimethoxy-5-hydroxyflavone, 3,6-dimethoxy-2-(3′,5′-dimethoxy-4′-hydroxyphenyl)-8,8-dimethyl-4H,8H-benzo[1,2-b:3,4-b′]dipyran-4-one, pongaflavone, 5-hydroxy-3,6,7-trimethoxyflavone, 4′-*O*-methylluteolin 7-glucoside, quercetin 3-*O*-glucosylglucoside [44,45];
Naphtoquinone: 2,6′-bis-7-methyljuglone [43];
Phenolic acid: gallic acid [32];
Fats: glycerides of myristic, palmitic, stearic, oleic and palmitoleic acids [46];
Tannins, free sugars and proteins [30,32,42,43,44,46];
The fruits have been reported to be rich in vitamin C; 229 mg/100 g fresh fruit [47].

*Seeds*:

Triterpenoid: betulinic acid [32].

*Roots*:

Flavonoids: 5,7,3,5′-tetrahydroxy-3′methoxyflavanone-4′-*O*-α-L-rhamnopyranoside, 5,7,3′,4′-tetrahydroxyflavanone-3-*O*-β-d-glucopyranosyl(1→4)-α-l-rhamnopyranoside [48,49].

**Figure 2:**
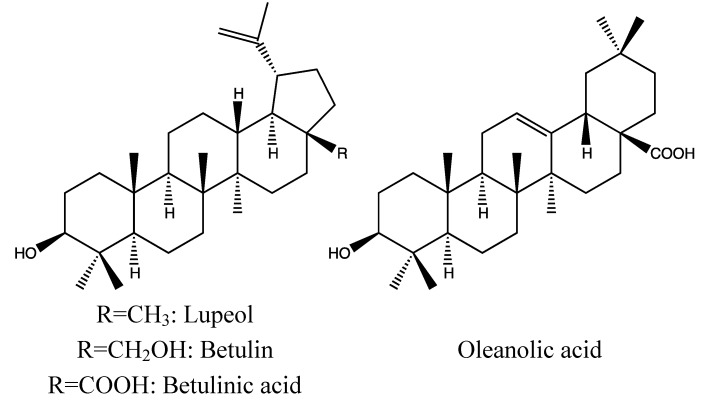
Triterpenoids from *Diospyros peregrina* bark and seeds. In the formulas in this paper, selected substances from the active plant parts are shown. Ubiquitous substances have been omitted. In most cases, it is unknown which substances are responsible for the anti-diarrhoea activity.

#### 2.1.3. Bioactivity

The methanol extracts of the bark and the seeds of *D. peregrina* have been investigated as an antidiarrhoeal [50]. Mice received bark and seed extracts at oral doses of 250 and 500 mg/kg. Then diarrhoea was induced by oral administration of 0.5 mL castor oil 45 min after each treatment. Both the total number of faecal output and the total number of diarrhoeic faeces the next 4 h were significantly reduced for both dosages of seed and bark extract. In the gastrointestinal motility test mice received the same dosage as described above, and after 30 min they were fed with 1 ml of a charcoal suspension. After 30 min the mice were sacrificed in order to measure the intestinal movement. The results showed that *D. peregrina* extracts delayed gastrointestinal transit of charcoal significantly compared to the control. The seed extract showed stronger effect than the bark extract.

Anthelmintic activity of a methanol extract of unripe fruits of *D. peregrina* has been reported [51]. The methanol extract of *D. peregrina* fruits has shown inhibition of the growth of a number of bacteria [52]. The disc-diffusion and tube dilution methods were employed. *E. coli* was highly sensitive against the extract with a MIC value of 10 μg/mL. The extract also inhibited the growth of *V. cholerae* (100 μg/mL), different *Shigella* species (200 μg/mL), *Pseudomonas aeruginosa* (200 μg/mL), and *Staphylococcus aureus* (100 μg/mL). The antibacterial potential of the bark and seed methanol extracts were evaluated against pathogenic bacteria responsible for causing diarrhoea and dysentery by using the antimicrobial disc-diffusion test. The bark extract (600 μg/disc) inhibited the growth of *S. aureus*, *Shigella dysenteriae*, *E. coli* and *P. aeruginosa*, whereas the seed extract inhibited *S. aureus*, *S. dysenteriae* and *E. coli* [50]. 

In a series of papers, the antidiabetic and antioxidant effects of *D. peregrina* fruit extract have been investigated by Dewanjee *et al*. [45,53,54,55,56,57]. Inhibition of α-amylase, reduction of glucose uptake and radical scavenging properties have been suggested as possible mechanisms for the antidiabetic effect. Hypoglycemic and antihyperglycemic activity of aqueous fruit extract from *D. peregrina* has been reported [58].

A bark extract of *D. peregrina* was found to have antitumour activity against Ehrlich ascites carcinoma in mice [59]. The gum from the fruits of *D. peregrina* has recently been suggested to be useful as a binder in tablet formulation [60].

#### 2.1.4. Toxicity

Approximate LD_50_ value of *D. peregrina* extract (whole plant) in mice was found to be 2.6 g/kg (p.o.) [61]. 

#### 2.1.5. Comments

The use of bark against diarrhoea may be explained by the tannins present having astringent properties. The chemical composition of the bark methanol extract tested in the mouse model was not described, but it is likely to contain tannins, elsewhere reported in *D. peregrina* bark [58]. This extract had positive effect on chemically induced diarrhoea on mice and also showed some antibacterial activity, which are promising results. The seed extract showed even better effects in the mouse model, but since little is known about chemical composition and toxicity seeds are hard to recommend on a scientific basis. The methanol extracts of bark and seeds have shown antidiarrhoeal activity in mice and antibacterial effect against several bacterial species associated with diarrhoeal diseases. In several studies composition of different plant parts has been investigated, and flavonoids, triterpenoids and tannins have been reported repeatedly. More studies of the bark and seeds are, however, needed in order to identify the bioactive compounds. It seems reasonable, though, that the flavonoids and tannins in the bark and seeds may contribute to the medical effects. 

### 2.2. *Heritiera littoralis* Dryand (Sterculiaceae)

**Figure 3:**
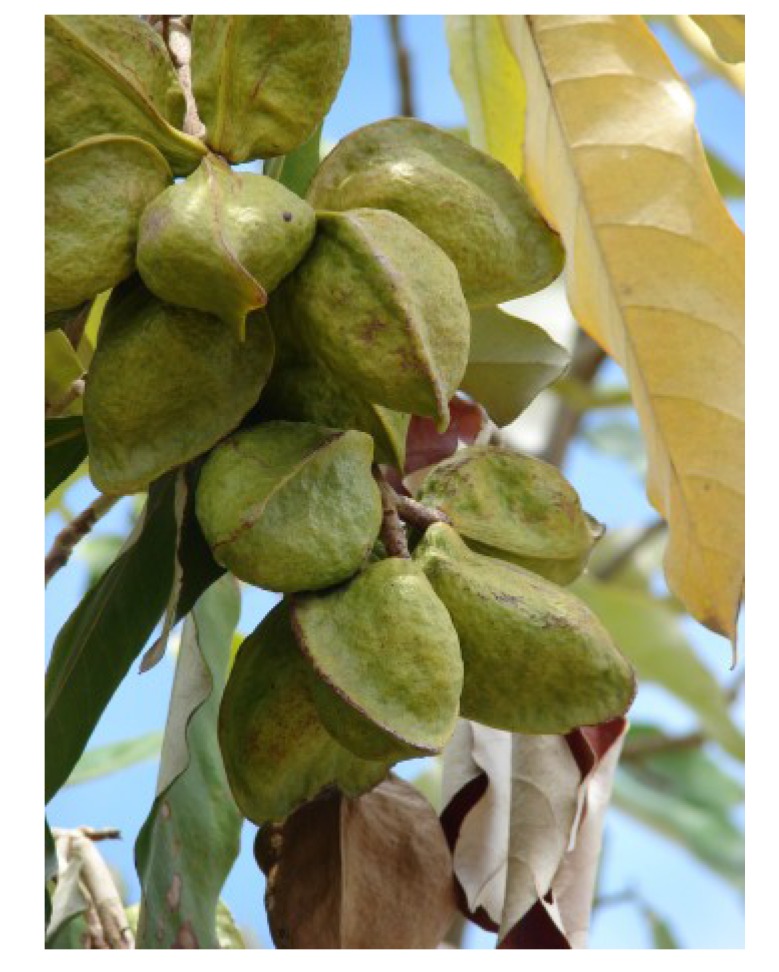
*Heritiera littoralis* [63].

*Heritiera littoralis* (dungun, looking-glass mangrove; Figure 3) is an evergreen mangrove tree, up to 25 m in height and with a buttressed trunk up to 60 cm in diameter. The bark is fissured, dark or gray. Leaves are 10–20 cm long, and they have a green upper surface and a silvery-white lower surface. The tree has numerous small bell-shaped, yellowish-green flowers. The fruits are hard and shining, 4–8 cm long [62]. *H. littoralis* is distributed from Madagascar and East Africa to Hong Kong, the Pacific and Australia. 

#### 2.2.1. Traditional Use and Plant Parts Used

*H. littoralis* seed extracts are traditionally used to treat diarrhoea and dysentery [64]. The stems and leaves have also been used against diarrhoea and dysentery. In addition, they have been used to control mosquitos and as a piscicide [65,66,67]. The sap is reported to be a fish poison and arrowhead and spearhead poison [64,68]. The seeds and leaves are, however, regarded as edible in the Andaman and Nicobar islands [69]. The tree is used as tooth brushes and chew sticks. The wood is also valuable for its timber [70].

#### 2.2.2. Chemical Composition (Figure 4)

*Bark*:

Coumarin: isofraxidin [64];
Triterpenoids: friedelin, betulinic acid *etc.* [64];
Sterols: β-sitosterol, stigmasterol, sitost-4-en-3-one, ergosterol peroxide, *etc.* [64];
Anthraquinone: physcion [64,71];
Tannins: [72].

*Leaves*:

Flavonoids: quercitrin, quercetin, kaempferol-3-*O*-(6″-*O*-*E*-p-coumaroyl)-β-d-glucopyranoside, kaempferol, kaempferitrin, myricetin, eriodictyol, afzelin, astragalin, tribuloside, catechin [73];
Lignan: isolariciresinol-3a-*O*-β-d-glucoside [74];
Others: (*Z*)-3-hexenyl β-d-glucoside, Me [β-d-xylopyranosyl-(1→6)-β-d-glucopyranosyl]-salicylate, and 2-*O*-[4′-(3″-hydroxypropyl)-2′,5′-dimethoxyphenyl]-1-*O*-β-d-glucopyranosyl-glycerol [73,74].

*Seeds*:

Fatty acids: malvalic, sterculic, palmitic, oleic and linoleic acid [75].

*Roots*:

Sesquiterpenes: heritol, heritonin, heritianin, vallapin, vallapianin [68,76,77,78];
Triterpenoid: friedelin [77].

**Figure 4:**
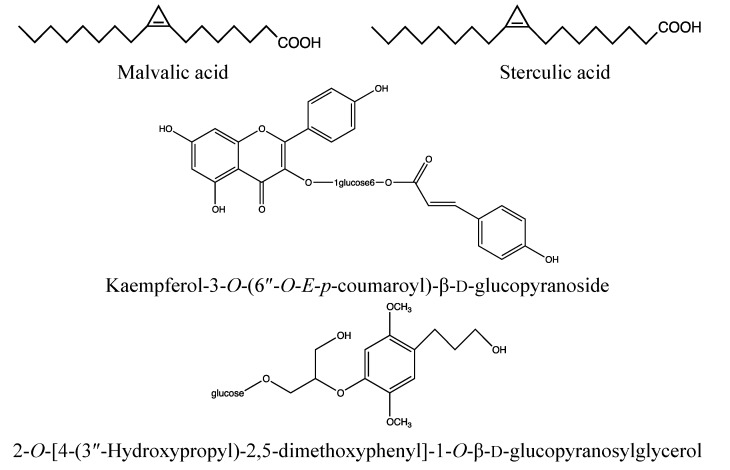
Fatty acids and phenolic constituents of *Heritiera littoralis* leaves and seeds.

In a related species, *Heritiera fomes*, we have found a high content of proanthocyanidins in the bark [79]. *H. fomes* is used locally in the Sundarbans against diarrhoea, accordingly to unpublished field work done by one of the authors of this review (*M. Alamgir*).

#### 2.2.3. Bioactivity

In spite of the often reported antidiarrhoea use of *H. littoralis*, it appears that no systematic scientific studies have so far evaluated the antidiarrhoeal effects of the plant. Aqueous leaf and stem extracts of the plant have shown antibacterial activity against *Salmonella paratyphoid*, while the ethanol extract was inactive. Some other bacteria, e.g., *S. aureus* and *P. aeruginosa* were also inhibited [80]. Several triterpenoids and steroids showed anti-inflammatory activity determined as NO inhibitory effect and anti-PGE_2_ activity, with ergosterol peroxide being the most active substance [71].

#### 2.2.4. Toxicity

The toxicity of *H. littoralis* to land animals has, as far as we know, not been evaluated. The sesquiterpenes from the roots are toxic to fish [68,77,78].

#### 2.2.5. Comments

There are no studies available on the efficacy of *H. littoralis* against diarrhoea, and toxicity testing has not been performed. However, since the stems are commonly used for maintaining dental hygiene and since leaves and seeds are regarded as edible, they can probably be considered as quite safe. Since no biological studies have been carried out to evaluate the chemical composition of seeds and few studies on pharmacological properties have been conducted we can only invite the scientific community to investigate this plant further.

### 2.3. *Ixora coccinea* L. (Rubiaceae)

**Figure 5:**
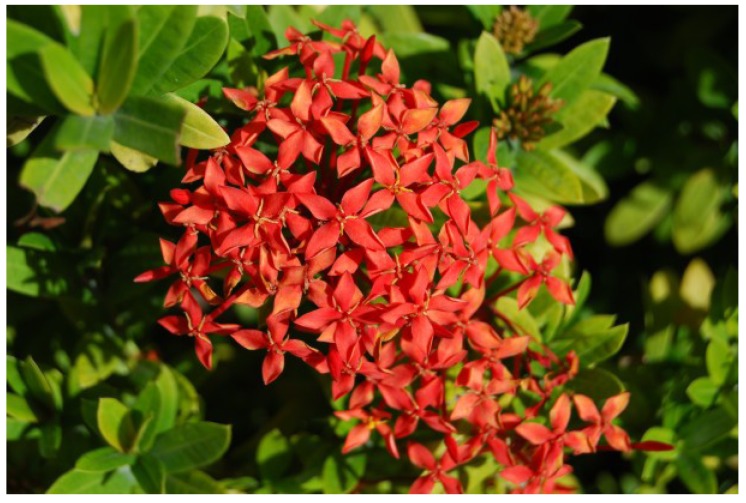
*Ixora coccinea* [82].

*Ixora coccinea* (Jungle geranium, Bengali: Kangan; Figure 5) is a perennial shrub 0.6–0.9 m in height, widely grown in gardens as an ornamental. The flowers are bright scarlet red, sometimes yellow, pink or orange-yellow. The bush has small globular fruits which are purple when ripe. The shrub is native to tropical Asia. However, it is cultivated for ornamental purposes in tropical and subtropical areas in other continents, as well [28,81].

#### 2.3.1. Traditional Use and Plant Parts Used

The roots, bark, leaves and flowers are used in traditional medicine in South East Asia from India to the Philippines [83,84,85,86]. The roots of *I. coccinea* are used to treat hiccoughs, nausea, fever, ulcers, gonorrhea, and loss of appetite. The flowers of *I. coccinea* are used against reddened eyes, eruptions, catarrhal bronchitis, dysentery, and as an anti-inflammatory agent. The leaves have been utilized in the treatment of diarrhoea. A paste from the root of an unspecified *Ixora* species is used against diarrhoea in children [87]. The ethnomedical uses and pharmacology of this plant have been reviewed previously [81,88].

#### 2.3.2. Chemical Composition (Figure 6)

*Leaves*:

Triterpenoid: lupeol [81,89];
Proanthocyanidins: ixoratannin A-2 (a trimeric A-type proanthocyanidin), procyanidin A2, cinnamtannin B-1 [90];
Flavonoids: epicatechin, kaempferol- and quercetin-rhamnosides [90].

*Flowers*:

Triterpenoids: ursolic acid, cycloartenol esters, lupeol esters, lupeol, oleanolic acid [84,91];
Sterol: sitosterol [84];
Flavonoids: biochanin A, myricetin, quercetin, rutin, daidzein formononetin, monoglycosides of cyanidin and delphinidin, rutin, kaempferol-3-rutinoside, traces of leucocyanidin glycoside [91].

*Above-ground parts*:

Triterpenoids: lupeol, 3-acetylbetulic acid, betunolic acid, α-amyrin, β-amyrin, ursolic acid, 3-acetylursolic acid, oleanonic acid [92];
Sterols: 6β-hydroxystigmast-4-en-3-one, sitosteryl-3-*O*-β-d-glucoside, β-sitosterol, stigmasterol [92];
Flavonoids: kaempferol, kaempferol-7-*O*-α-rhamnoside, kaempferitrin, luteolin, (−)-epicatechin, (+)-catechin [92];
Proanthocyanidin: epicatechin-4β->8, 2β->*O*->7-ent-epicatechin [92];
Coumarins: scopoletin, coumarin, *erythro*-1′,2′-albiflorin [92];
Diterpenoids: 16a-hydro-19-acetoxy-(−)kauran-17-oic acid, 16a-hydro-19-ol-(−)-kauran-17-oic acid [92];
Quinones: 1,4-dihydroxy-3-methylanthraquinone, tocopherylquinone [92];
Peptides: ixorapeptides I and II [92].

*Roots*:

Fatty acids: palmitic, stearic, oleic and linoleic acid [93];
Essential oil: (main constituent β-sesquiphellandrene) [81,94].

**Figure 6:**
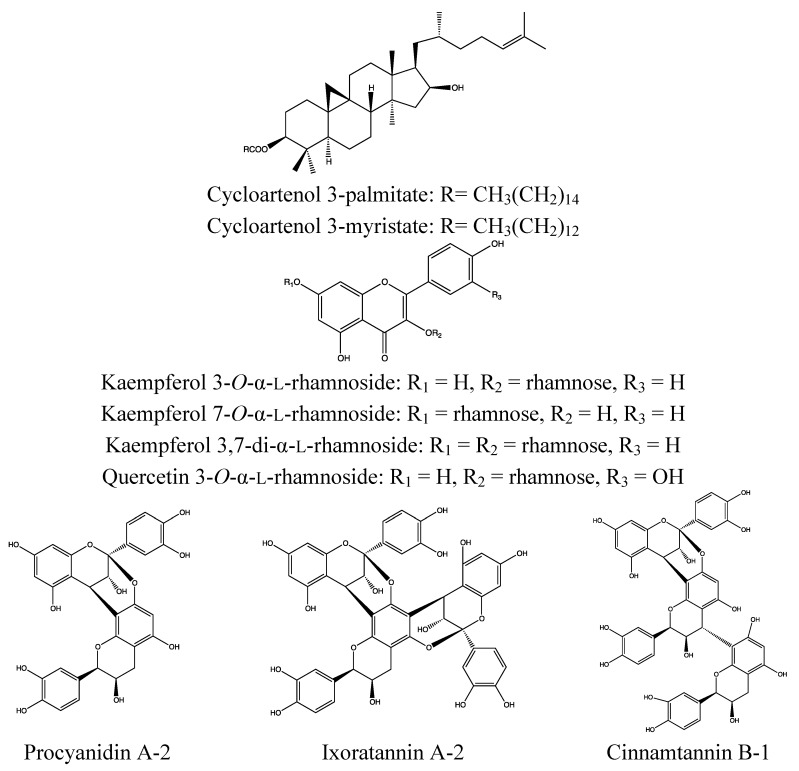
Triterpene esters and polyphenols from *Ixora coccinea*.

#### 2.3.3. Bioactivity

The antidiarrhoeal effect of *I. coccinea* has been investigated [95]. An aqueous extract of the flowers showed significant inhibition of castor oil induced diarrhoea in rats as determined by weight and volume of intestinal content and by gastrointestinal motility.

*I. coccinea* has been investigated for antimicrobial effects. In a study by Annapurna *et al.* [96], ether and methanol extracts of the leaves were tested against a selection of bacteria and fungi. The ether extract was found to have higher activity than the methanol extract, and both Gram-negative and Gram-positive bacteria were inhibited. The activity against fungi was not significant [96]. Srinivasan *et al*. [94] reported that the essential oil from *I. coccinea* roots exhibited antimicrobial activity towards Gram-positive and Gram-negative bacteria. Sasidharan [97] found that an alcoholic extract of *I. coccinea* (plant part not specified) was active against *S.* *aureus* and *E. coli*, the aqueous extract was active against *E. coli* but not *S. aureus*, and neither the aqueous nor the alcoholic extract were active against the fungi *Aspergillus niger*. Leaf constituents were active against *E. coli*, *S. aureus*, *P. aeruginosa* and *B. subtilis* [90]. In these studies the disc-diffusion method was employed. Ethanolic and aqueous root extracts inhibited bacterial growth of *Enterococcus faecalis*, *E. coli*, *Salmonella typhi* and several other bacteria (*S. aureus*, *B. pumilus*, *P. aeruginosa*) with MIC values of 12.5–100 μg/mL. The extracts were, however, inactive against fungi. Interestingly, these extracts were also reported to have wound-healing properties [98]. 

An ethanol extract of *I. coccinea* roots protected rats from aflatoxin B1-induced liver damage. This was suggested to be due to the potent antioxidant activity of the extract [99]. Antioxidant activity of leaf constituents were reported by Idowu *et al.* [90]. Antioxidant properties (as radical scavenging, total antioxidant capacity, and xanthine oxidase inhibition) of methanol extracts from flowers, leaves and stems of *I. coccinea* were reported [100]. The antioxidant activity seemed to be correlated to the phenolic content. The antioxidant properties of the methanolic extract of *I. coccinea* was also believed to be important for its ability to counteract doxorubicin induced cardiotoxicity in rats [101]. 

Haridass *et al.* [102] investigated the antioxidant and cytotoxic activity of petroleum ether, ethyl acetate and methanol extracts of *I. coccinea* flowers, finding that the ethyl acetate extract was the most active one. Ixorapeptide I was found to have selective cytotoxicity towards Hep3B liver cancer cells relative to normal cells [92]. An aqueous extract of the flowers of *I. coccinea* had antimutagenic activity [103]. 

An aqueous extract of the leaves exhibited hypoglycaemic and hypolipidaemic activity in diabetic rats [104]. A methanolic flower extract had anti-inflammatory and analgesic properties [105].

The methanol extract of *I. coccinea* leaves has been reported to be without larvicidal activity towards *Anopheles* mosquitoes [106].

#### 2.3.4. Toxicity

In a mice toxicity test it was found that the petroleum ether extract of *I. coccinea* root, up to an oral dose of 1.5 g/kg body weight, did not show any toxic effects [107]. In another study, the active fraction (AF) (the cytotoxic fraction from a flower hexane extract) up to 400 mg/kg was given i.p. to mice. No deaths were observed in 24 h [108]. The test animals did not show any changes in general behavior during the study. Chronic administration of AF (200 mg/kg i.p.) did not produce any significant differences in the food or water consumption and body weight of the mice either. However, Atiq-Ur-Rahman *et al.* [109] have reported that a methanolic extract of *I. coccinea* flowers and fractions therefrom were cardiotoxic to the perfused guinea pig heart and might lead to heart failure. Whether this is relevant for *in vivo* conditions appears unknown.

#### 2.3.5. Comments

The leaves of *I. coccinea* have been used in traditional medicine against diarrhoea. This activity has recently been documented in animal experiments. Numerous biological activities have been reported for different parts of the plant, although most of these are *in vitro*. It would appear that some of these effects are related to the antioxidant activity of the plant, which again has been suggested to be correlated to its content of phenolic compounds such as flavonoids and A-type proanthocyanidins. In this connection, it might be mentioned that A-type proanthocyanidins from cranberries have been reported to be partly responsible for the putative effects of cranberries against urinary bladder infections [110]. Investigation of proanthocyanidins from *I. coccinea* for this condition might seem interesting.

### 2.4. *Pongamia pinnata* (L.) Pierre (Fabaceae)

**Figure 7:**
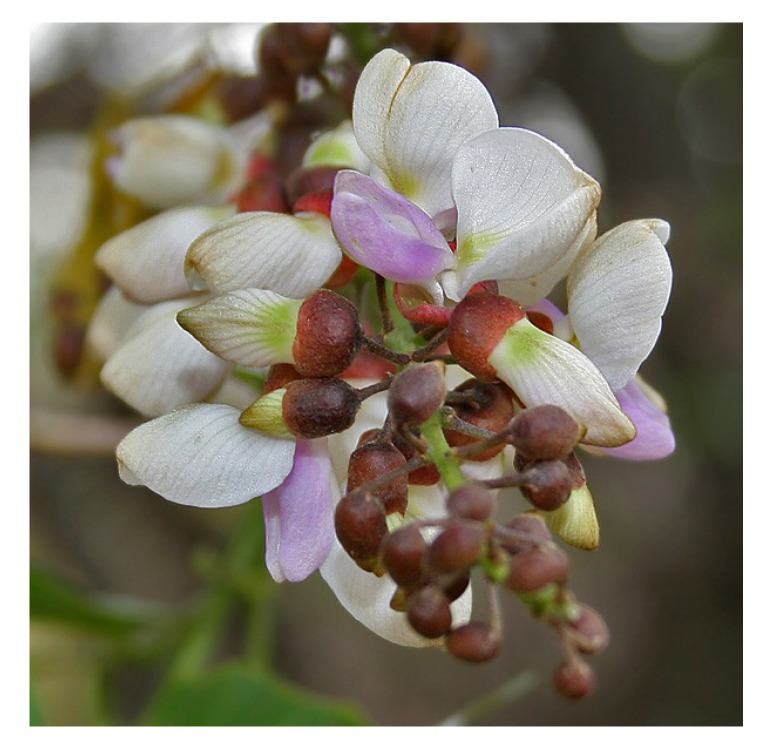
*Pongamia pinnata* [115].

*Pongamia pinnata* (syn. *Pongamia glabra* Vent.; Figure 7) is a medium sized tree, 15–25 m in height, with white, purple and pink flowers growing in clusters and maturing into brown seed pods. The species is distributed from India to Philippines and the north of Australia. “Karanja” is the local name used in Bangladesh [28]. The traditional use, chemistry and pharmacology of this tree have been reviewed [111,112,113,114].

More than a thousand scientific articles, as indexed in the SciFinder database, deal with *Pongamia pinnata* and its constituents, and it would be beyond the scope of this article to give a detailed review of this plant. In recent years, a large number of papers have dealt with *P. pinnata* as a source of biofuel. These are not covered in the present paper.

#### 2.4.1. Traditional Use and Plant Parts Used

*P. pinnata* has been widely used as a traditional medicinal agent, and both the leaves, bark, flowers, seeds, and roots are reported to have a healing effect [28,30,114]. The leaves of *P. pinnata* have been used against flatulence, dyspepsia and diarrhoea [86]. In Tamil Nadu, India, preparations of the plant are used against gastric trouble and as a cure for wounds [116,117]. A poultice of the leaves is applied to ulcers infested with worms. A decoction of the leaves is used for medicated baths and fomentations in cases of rheumatic pains. The juice from the roots is used for closing fistulous sores and for cleaning foul ulcers. It is used for cleaning the teeth and strengthening the gums. It is also given internally mixed with coconut milk and lime water for the cure of gonorrhea. The oil from the seeds is useful in skin diseases such as herpes and scabies, and in rheumatism [86]. A paste from the seeds has also been used in rheumatism [118]. The use of the bark or leaves of the plant against fever in humans [119] and animals [120] and against malaria [69] has been reported. Both the seeds and roots are used as fish poison. The fresh bark is used internally in the treatment of bleeding piles [86]. In Madhya Pradesh, India, the plant has been used against burns, but no details are given [121]. It is also reported that the plant is recommended for the treatment of snake bites and scorpion stings. However, the efficacy of this treatment has been debated [28,30]. 

#### 2.4.2. Chemical Composition (Figure 8)

*P. pinnata* is rich in flavonoids, especially prenylated flavonoids which are common in the Fabaceae family. A search in the SciFinder database with the keyword “*Pongamia pinnata* flavonoid” resulted in 100 hits, and several hundred different chemical constituents are reported in the literature. To limit the number of chemical constituents listed for this plant, this paragraph will focus on the chemistry of the leaves, which are the plant parts used for diarrhoeal diseases.

*Leaves*:

Flavonoids: [122,123,124,125,126,127];
Chromenes: glabrachromene I and II [124];
Triterpenoids: cycloart-23-ene-3β,25-diol, friedeline, lupeol, lupenone, betulin [126,127];
Sterol: β-sitosterol [124,126];
Fatty acids: [126].

**Figure 8:**
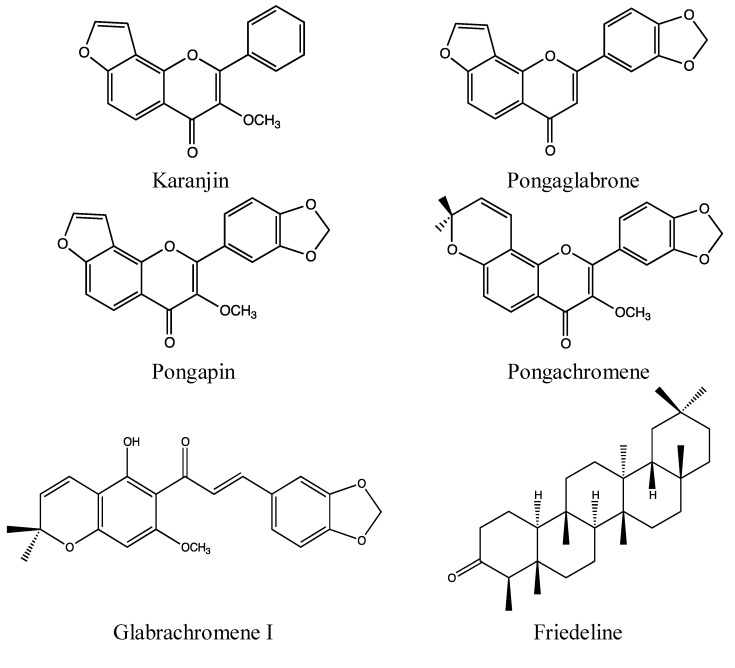
Flavonoids, a chromene and a triterpene from *Pongamia pinnata* leaves.

#### 2.4.3. Bioactivity

Until now, two studies have investigated the antidiarrhoeal activity of *P. pinnata* leaves. In the first study, the methanol leaf extract was given to mice in doses of 3, 7.5 and 15 mg/kg, 30 min before castor oil administration. The mice were observed the next four hours, and it was found that the onset time of diarrhoea was significantly increased, and the total number of faeces, the number of wet faeces and the total weight of wet faeces were significantly reduced when compared to control group [128]. In a study by Brijesh *et al*. [129], the antidiarrhoeal properties of hot water decoction of *P. pinnata* leaves were investigated against various virulence parameters of infectious diarrhoea. The decoction showed no activity against *Shigella flexneri*, *V. cholerae* or of different strains of *E. coli.* The viability of *Giardia lamblia* trophozoites and rotavirus were not affected. However, the production of cholera toxin was significantly reduced when *V. cholerae* was grown in the presence of the decoction (1%, 5% and 10%). The production of labile toxin by *E. coli* was not affected either. Finally, the decoction did not inhibit the adherence of *E. coli* to epithelial cells. However, the invasion of both *E. coli* and *S. flexneri* into epithelial cells was significantly reduced after treatment with the leaf decoction. Antibacterial activity of leaf extracts *in vitro* has been reported by several groups [130,131,132,133,134,135,136]. Most of these investigations include enteric bacteria such as *E. coli*, *Salmonella typhimurium*, *S. typhi*, and *Enterobacter aerogenes*. Interestingly, activity against methicillin-resistant *S. aureus* was observed by Ramesh *et al.* [132]. Antioxidant properties of leaf extracts have been reported [137,138].

Numerous other activities have been reported for extracts and constituents from *P. pinnata.* A few examples are: Antidiabetic activity of leaf extract of *P. pinnata* and of cycloart-23-ene-3β, 25-diol has been investigated [138,139,140,141]. This compound has also been shown to have antioxidant and antimicrobial properties [142]. The flavonoids pongamol and karanjin from *P. pinnata* also have been reported to have antihyperglycemic effect, apparently by regulating the levels of the insulin-sensitive glucose transporter GLUT4 [143,144,145]. The methanolic seed extract [146] and the substance karanjin [147] has been reported to exert a gastroprotective activity *in vivo* in rats. Anti-dyslipidemic and antioxidant activities of fruit extracts were investigated by Bhatia *et al.* [148].

Anti-insect activity of *P. pinnata* has been reported repeatedly. Effect against larvae of the mosquitoes *Aedes aegypti* and *Culex quinquefasciatus* has been shown [149,150,151]. In addition, oviposition deterrent activity towards these mosquitoes was found by Swathi *et al.* [152]. A petrol ether extract of the leaves showed promising activity as a pediculocide aginst head lice, *Pediculus humanus capitis* [153].

A rather unusual preparation of *P. pinnata* was reported by Shanthi *et al.* [154]: leaves extracted in cow urine were reported to be a remedy for bacterial leaf blight in rice paddies.

#### 2.4.4. Toxicity

The ethanol extract of the leaves was administered orally to mice in doses of 3.0–10.125 g/kg [155]. In the next 24 h no toxic symptoms or mortality were observed. In another study, no toxic effects were observed in mice after dosages of 250 mg/kg of methanol leaf extract [156]. An ethanolic flavonoid extract of the leaves was non-toxic in human erythrocytes *in vitro* [135], and pongamol, an important bioactive constituent of the plant, appeared non-toxic towards rats [156].

#### 2.4.5. Comments

The leaves of *P. pinnata* have been used in the treatment of diarrhoea. The presented studies indicate a potential antidiarrhoeal effect of the leaves. Until now, only the low-molecular weight compounds of the leaves have been systematically described, and they comprise a number of methoxylated furanoflavones. However, the chemistry of the aqueous extract investigated by Brijesh *et al.* [129] has not been studied in detail. The plant leaves seems to have low toxicity. More studies are needed to clarify the mode of action and to identify the constituents responsible for the anti-diarrhoeal properties. The flavonoids in the plant might be correlated to this activity, although numerous other constituents have been described. In sum, this plant appears to be of high interest, both from a pharmacological and a phytochemical point of view.

### 2.5. *Rhizophora mucronata* Lamk. (Rhizophoraceae)

**Figure 9:**
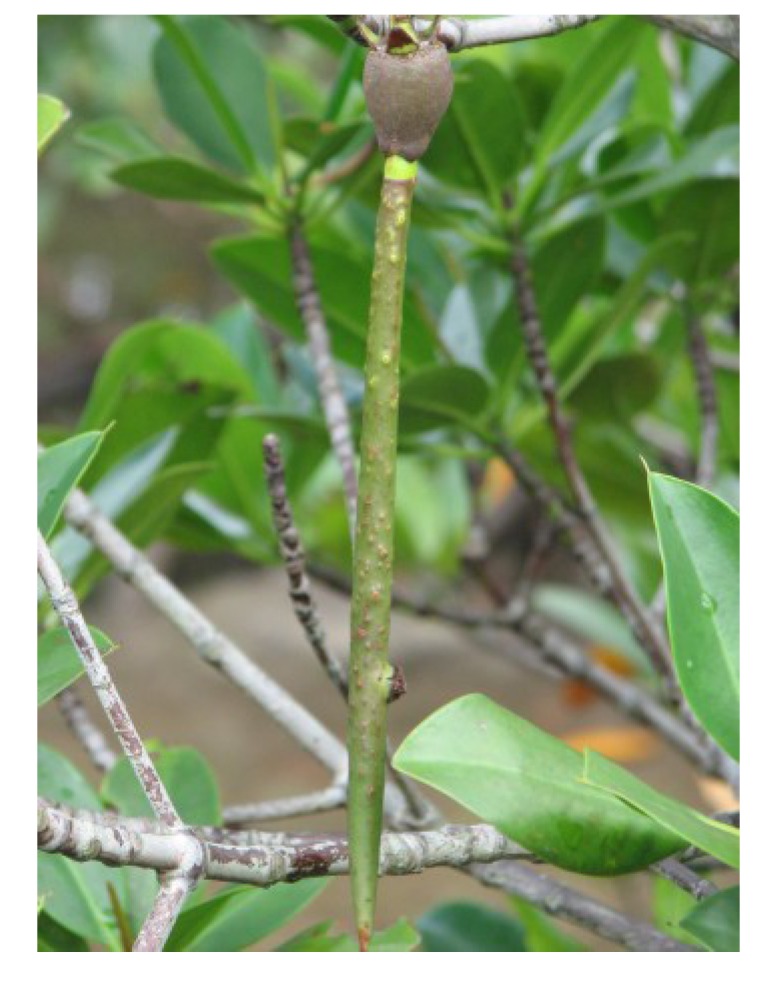
*Rhizophora mucronata* [157].

*R. mucronata* (Figure 9) is an evergreen small tree up to 15 m tall, with small white flowers and long ovoid-conical fruits. The tree is distributed along muddy shores and tidal creeks in tropical zones of East- and South Africa, Asia, Northeast Australia and Central America [28]. “Bhora” is the local Bangladeshi name.

#### 2.5.1. Traditional Use and Plant Parts Used

The bark is known as an astringent, and it has traditionally been used in the treatment of diabetes, diarrhoea, nausea, haematuria, haemorrhages and angina [28,86]. The bark has been used for extraction of tannins for the leather industry and for dyeing purposes which also continues today [64]. The traditional use of several mangrove plants including *R. mucronata* has recently been reviewed [158].

#### 2.5.2. Chemical Composition (Figure 10)

*Bark*:

Tannins (hydrolysable) [72,86,159,160];
Lignin [72,86];
Triterpenoids: Lupeol, β-sitosterol, adene-5-en-3-ol, 3β-*O*-(*E*)-4-methoxycinnamoyl-15α-hydroxy-β-amyrin [161,162,163];
Flavonoid: quercetin [161,163];
Phenolic acid: caffeic acid [161].

*Leaves*:

Indole: rhizophorine [164];
Diterpenoids: gibberelline A_3_, A_5_ and A_9_, phytol [165,166];
Triterpenoids: β-amyrin, α-amyrin, betulin, lupeol, ursolic acid, squalene [86,167];
Sterols: cholesterol, stigmasterol, β-sitosterol, campesterol, 28-isofucosterol, stigmast-7-en-3β-ol [86];
Fatty acids: [166,167,168];
1-hydroxy-5-oxobicyclo[6.4.0]dodecane [169];
Tannins [86] and catechins (flavonoids) [170] have been reported, as well.

*Fruits*:

Triterpenoids: 3β-*E*-caffeoyltaraxerol, 3β-*E*-p-coumaroyltaraxerol, 3β-*Z*-p-coumaroyltaraxerol, 3β-*Z*-caffeoyltaraxerol, β-taraxerol [171];
Sesquiterpenoid: mucronatone [171];
Unripe fruits are rich in tannins, the ripe ones are less so [86].

*Roots*:

Diterpenoids: rhizophorin A-D [172,173,174];
Sugar alcohol: 1d-1-*O*-methyl-*muco*-inositol [175];
Tannins: [160];
Lignin: [160].
  25*S*-spirost-5-ene-3β,14α-diol (steroid sapogenin) has been reported, plant part not specified [176].

**Figure 10:**
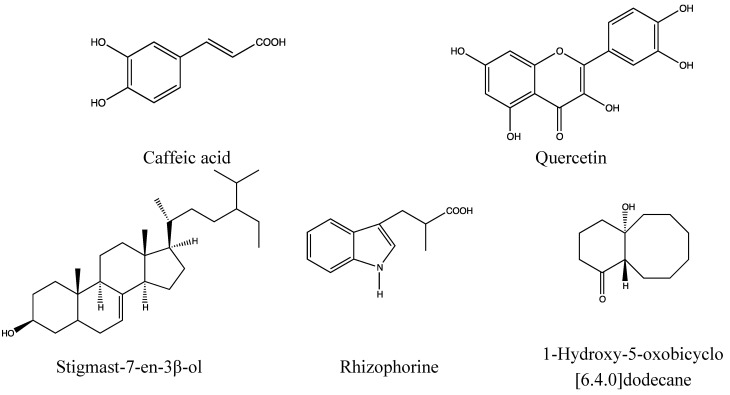
Constituents of *Rhizophora mucronata* bark (above) and leaves (below).

#### 2.5.3. Bioactivity

Antidiarrhoeal and antiinflammatory activity of *Rhizophora mucronata* bark extracts were reported by Rohimi and Das [161]. Of the substances isolated, quercetin and caffeic acid appeared to have highest activity as antidiarrhoeals. In addition, sitosterol, lupeol and adene-5-ene-3-ol were anti-inflammatory, but did not show antidiarrhoeal effect. Antidiarrhoeal effect was also demonstrated in a methanol extract of the leaves [170]. 

Antiviral activity has been investigated [177,178,179]. The ethanol bark extract was found to have high activity against the Newcastle disease, vaccinia, encephalomyocarditis and Semliki Forest viruses. Also the ethanol flower extract showed good activity, except against the vaccinia virus. A polysaccharide from the bark protected cells from HIV-induced cytopathogenicity *in vitro*. Among 73 extracts of marine plants and mangroves, the bark of *R. mucronata* was the most promising antiviral agent [179]. Leaf methanol extracts exhibited antibacterial activity towards drug resistant *Vibrio* spp. and *S. aureus* [180,181,182]. Hexane and chloroform extracts of leaves and roots of *R. mucronata* showed strong inhibitory activity towards a series of bacteria and fungi [183]. Ethanol-water extracts of the bark and flowers showed antiplasmodial activity towards *P. falciparum* with IC_50_ values of 62 μg/mL (bark), 92 μg/mL (flowers) [184,185].

Extracts of *R. mucronata* showed moderate antioxidant effect determined in the FRAP and DPPH assays [186,187].

#### 2.5.4. Toxicity

Honey from the flowers is reported to be poisonous [86]. Scientific toxicity studies have, as far as we know, not been carried out.

#### 2.5.5. Comments

The bark of *R. mucronata* seems to have widest application in traditional medicine of this plant, e.g., against diarrhoea. The few studies that have been performed seem to confirm the traditional use. It appears reasonable that tannins and other polyphenolics in the bark may be involved in the antidiarrhoeal properties. The antiviral, antibacterial and antifungal tests indicate promising bioactivity of the bark and leaves. Toxicity studies should also be performed.

### 2.6. *Xylocarpus granatum* König (Meliaceae)

**Figure 11:**
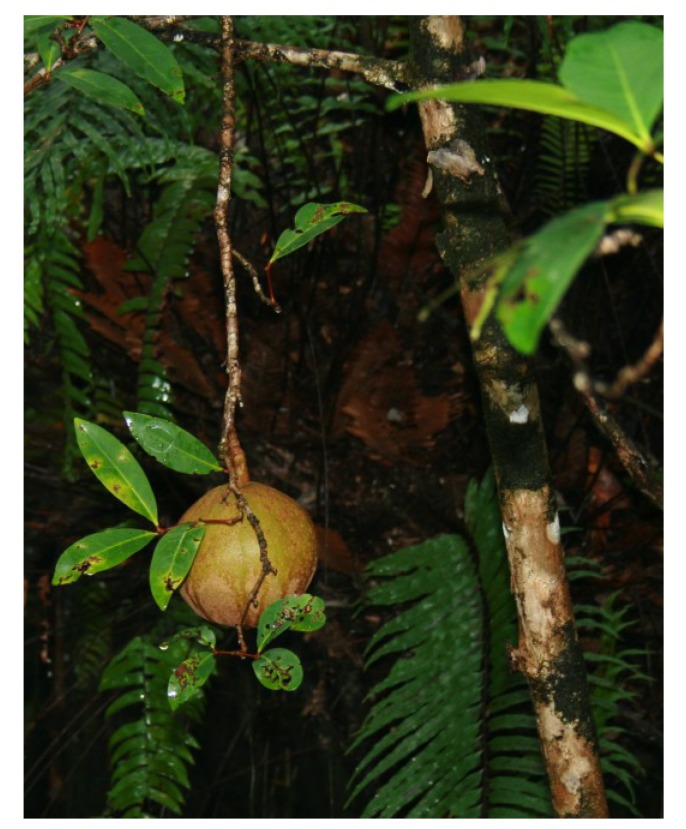
*Xylocarpus granatum* [189].

*Xylocarpus granatum* (Figure 11), also known as “dhundul” in Bangladesh, “cannonball tree” or “puzzle nut tree”, is an evergreen tree with gray bark, up to 15 m in height. The fruits can be up to 25 cm in diameter [62]. *X. granatum* is distributed in mangrove forests in East Africa, tropical Australia and Southeast Asia [62,188]. The bark possesses extreme bitterness [28].

#### 2.6.1. Traditional Use and Plant Parts Used

The astringent bark is used to treat fever, cholera, colic, diarrhoea and other abdominal affections [28,86,190,191]. To treat diarrhoea, the bark is used traditionally as a water decoction prepared overnight [192]. The bark has been used for tanning and for the preparation of dyes of umber color [193]. The fruits are also used against diarrhoea and externally to soothe inflammation [192,194]. An Indian patent application [195] describes the use of fruit seed coats of *X. granatum* for making an antidiarrhoeal drug. Seed kernels are used in tonics, and the bitter and astringent oily fluid from the seeds is taken for diarrhoea, dysentery, as an illuminant, and as hair oil [196].

#### 2.6.2. Chemical Composition (Figure 12)

*Bark*:

Flavonoids: catechin, epicatechin [197];
Proanthocyanidins: procyanidin B1 (epicatechin (4β → 8) catechin), procyanidin trimer and pentamer (composed of catechin as the starter and epicatechin as the extender units) [197,198];
Triterpenoids: gedunin, xyloccensins L–V, 6-dehydroxyxylocarpin D (limonoids) [197,198,199,200,201,202,203];
Condensed tannins: [72,86,160]. The bark is stated to contain more than 20% tannins [86].

*Wood*:

Triterpenoid: gedunin (limonoid) [204];
Tannins: (composition not specified) [160,204].

*Leaves*:

Sterols: cholesterol, campesterol, stigmasterol, sitosterol, 28-isofucosterol [167];
Hydrocarbons and fatty acids [167];
Long-chain aliphatic alcohol: triacontanol [205];
Flavonoid: kaempferol 3-*O*-β-d-glucoside [205];
Lactone: 3-(1-hydroxyethyl)-4,4-dimethyl-4-butyrolactone [205].

*Seeds*:

A large number of limonoids and protolimonoids (triterpenoids) have been described [190,206,207,208,209,210,211,212,213,214,215,216,217,218,219,220,221,222,223];
Sterols: ergosterol peroxide, β-sitosterol, daucosterol [216];
Triterpenoid: hispidol B [216];
Coumarin: scopoletin [216].

*Fruits*:

Triterpenoids: limonoids and protolimonoids [224,225,226,227,228,229,230,231,232,233,234], butyrospermol fatty acid esters [229];
Sterols: daucosterol [224], β-sitosterol fatty acid esters [229];
Peptide: aurantiamide [224];
Flavonoid: catechin [224];
Sesquiterpenoid: abscisic acid [224];
Benzoic acids: 4-hydroxybenzoic acid, ethyl 3,4-dihydroxybenzoate [224];
Alkaloids: xylogranatinin [234], granatoin [226];
α-Tocopherol (vitamin E) [224].

The chemistry of the genus *Xylocarpus* has been reviewed [235].

**Figure 12:**
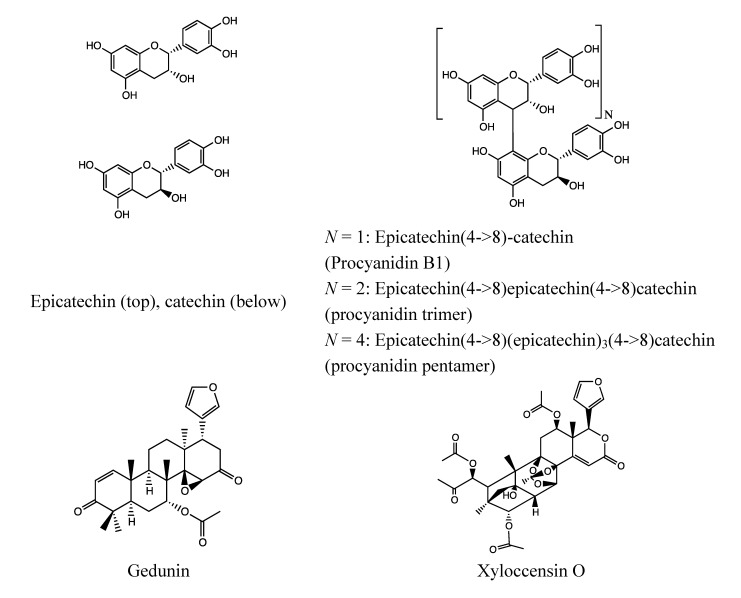
Catechins and proanthocyanidins (above) and limonoids (below) from *Xylocarpus granatum* bark.

#### 2.6.3. Bioactivity

To scientifically investigate the antidiarrhoeal effect of *X. granatum* bark, Rouf *et al.* [192] administered methanol extract of *X. granatum* bark to diarrhoeal induced mice and studied the effect on gastrointestinal motility. The results showed a significant dose-dependent reduction in the total number of faeces in 4 h after administration of 250 and 500 mg/kg methanol extract. The intestinal transit of charcoal meal after peroral administration of 250 and 500 mg/kg of the extract was significantly reduced as well. 

The methanol extract from *X. granatum* bark showed antibacterial action against *Kocuria rhizophilia*, *S. aureus*, *B. subtilis* and *P. aeruginosa* in the disc-diffusion assay [198]. Growth of *E. coli* and *C. albicans* was not affected. 

Ethanol extracts of pericarp and seeds of *X. granatum* inhibited the growth of the pathogenic bacterium *Acinetobacter baumannii* [236]. Extracts of the stem bark made with different solvents showed activity against *Staphylococcus aureus*, *S. epidermis*, *E. coli*, *Shigella boydii* and *Proteus sp.*, but were inactive towards *Shigella dysentery*, *Salmonella typhi* and *Enterococci* [237]. Xyloccensins isolated from *X. granatum* have been tested as antimicrobial agents. In the first study, the limonoid gedunin (0.5%–2.0% solutions of gedunin was impregnated into wood blocks) isolated from the stem was found to exhibit antifungal activity against the wood-rot fungi *Polyporus palustris* and *P. versicolor* [204]. 3-(1-Hydroxyethyl)-4,4-dimethyl-4-butyrolactone showed fungicidal activity towards powdery mildew [205]. Xyloccensin I, J, O and P were found to have no antimicrobial effects [190,198], and hexane leaf extracts and and methanol stem extracts of *X. granatum* were found inactive or nearly inactive towards a series of fish pathogenic bacteria [238].

Some of the limonoids have antifeedant effect towards larvae of *Mythimna separata* [219]. Gedunin and photogedunin had antifilarial activity towards the human parasite *Brugia malayi* [239], and a chloroform extract of the fruits of *X. granatum* showed antimalarial activity against *Plasmodium falciparum* [240]. The activity was ascribed to gedunin and xyloccensin-I.

Gedunin and photogedunin from *X. granatum* had anti-secretory effect and were protective against peptic ulcer in mice [241]. Gedunin also inhibited the growth of Caco-2 colon cancer cells *in vitro* [242].

#### 2.6.4. Toxicity

It is reported that the plant is used by the Orange-Dyakouns of the Malay-Peninsula to prepare their poison “ipokrohi” [28]. However, scientific toxicity studies have not been carried out on this plant. 

#### 2.6.5. Comments

The bark from *X. granatum* is widely used in tropical Asian countries as an antidiarrhoeal agent, and the pharmacologic effect of the methanolic bark extract has been demonstrated [192]. The methanol extract contains high amounts of flavan-3-ols and procyanidins [198]. Proanthocyanidin-rich plants have a long tradition of use as antidiarrhoeals in folk medicine [20], and this class of compounds may contribute to the observed effects. Inhibitory activity towards bacteria involved in gastrointestinal disease may also be of importance. Chemical studies of *X. granatum* have also shown a high variety of tetranortriterpenoids in lipophilic extracts of the seeds, fruits and stem bark. Until now, more than 50 limonoid derivatives have been identified. However, further studies are necessary both to identify the active antidiarrhoeal compounds and their mode of action. Additionally, controlled clinical studies are needed to evaluate the effects in humans. Finally, more toxicological data are necessary. 

### 2.7. *Xylocarpus moluccensis* M. Roem (Meliaceae)

**Figure 13:**
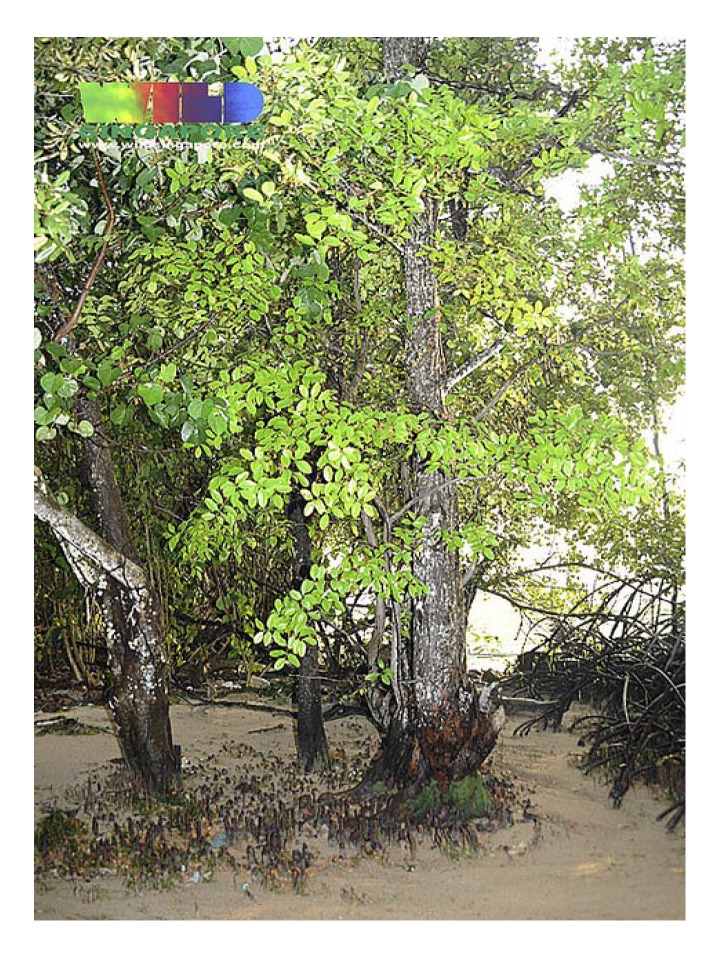
*Xylocarpus moluccensis*. Note: this picture is reproduced with permission of Ron Yeo. Copyright © 2013 Ron Yeo (Ron Yeo@tidechaser.blogspot.com).

*Xylocarpus moluccensis* (Figure 13) is a glabrous, medium sized tree that grows in the tropical mangroves spanning from East-Africa to Philippines, Australia and the Pacific Islands [62]. In Bangladesh, the tree grows in the north tract, remote from the sea, chiefly in the low lying swampy locality of the Sundarbans. The local name in Bangladesh is “passur” [243].

#### 2.7.1. Traditional Use and Plant Parts Used

The bark is used as an astringent and a febrifuge, and has been used traditionally in the treatment of fever, dysentery, diarrhoea and other abdominal troubles [244]. Fruits from *X. moluccensis* have been used to cure swellings of the breast and elephantiasis [28,243] and (powdered or as a decoction) against diarrhoea [245]. An ointment prepared from seed ash, sulphur and coconut oil is used as a cure for itch [246].

#### 2.7.2. Chemical Composition (Figure 14)

*Bark*:

Flavonoids: catechin, epicatechin [197];
Proanthocyanidins: procyanidin B1 (epicatechin (4β → 8) catechin), procyanidin B3 (catechin (4α → 8) catechin), procyanidin trimer, procyanidin pentamer, procyanidin hexamer, procyanidin decamer and procyanidin undecamer (non-hydrolyzable tannins) [197].

*Wood*:

Triterpenoids: limonoids [247,248,249];
Fatty acid: 4-oxo-19-phenylnonadec-5-enoic acid [250].

*Fruits*:

Triterpenoids: limonoids [251];
Monoterpenoid: (secoiridoid): xylomollin [252].

*Seeds*:

Triterpenoids: limonoids [190,247,248,253,254,255,256,257,258,259,260,261], tirucallane-type triterpenoids [260];
Protein, minerals and fatty acids [262].

**Figure 14:**
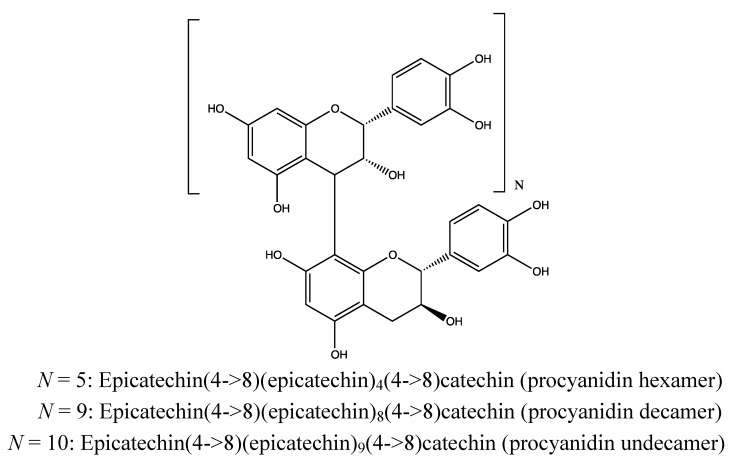
Proanthocyanidins from *Xylocarpus moluccensis* bark.

#### 2.7.3. Bioactivity Studies

In castor oil- and magnesium sulphate-induced diarrhoeal mice the methanol bark extract of *X. moluccensis* (250 and 500 mg/kg) reduced the severity of diarrhoea dose-dependently [244]. The methanol fraction (500 mg/kg) did also reduce the intestinal transit of charcoal meal in mice. In the same study, the ethyl acetate and residual methanol fractions at 250 mg/kg showed an even stronger antidiarrhoeal effect while the chloroform soluble fraction was inactive. 

The antibacterial effects of the methanol crude extract, the chloroform and ethyl acetate soluble fractions and the residual methanol fraction have been investigated [244]. The disc-diffusion method was employed with 500 μg of extract per disc. The methanol crude extract was active against *E. coli*, *V. cholerae*, *S. dysenteriae*, *S. aureus*, *Staphylococcus epidermis*, *Staphylococcus pyogenes*, *Salmonella typhi*, *P. aeruginosa* and *Enterobacter aerogenes*. The growth of *Shigella boydii*, *S. flexneri* and *Shigella sonnei* were not affected. The tested bacteria are associated with diarrhoea and dysentery.

The limonoids of *X. moluccensis* seeds have been investigated for insecticidal, pesticidal and anti-feedant effects, with many of them being found active [254,256,258,260,261]. Some of these applications have been patented [263,264]. 

7-Deacetylgedunin from *X. moluccensis* seeds has anti-inflammatory activity [259], and 7-oxo-deacetoxygedunin inhibits osteoclastogenesis and has been suggested as a possible treatment for osteoporosis [265].

Methanolic extracts of bark and pneumatophores of *X. moluccensis* had CNS depressant properties, with the pneumatophore extract showing the highest activity [246].

#### 2.7.4. Toxicity

Methanolic and aqueous extracts of *X. moluccensis* pneumatophores showed cytotoxicity towards two human cancer cell lines, but were inactive against normal mouse fibroblast cells [243].

#### 2.7.5. Comments

*X. moluccensis* bark has shown significant antidiarrhoeal effect in mice models. The mode of action may be exerted by antisecretory mechanisms, by preventing the reabsorption of water and by delaying the gastrointestinal transit, and by a direct antibacterial activity. The traditional use of bark from *X. moluccensis* as an antidiarrhoeal agent can likely be ascribed to its content of flavan-3-ols and procyanidins which are found in high content in the methanol crude extract and in fractions that are polar and semipolar. *X. moluccensis* is rich in lipophilic limonoids. Since the chloroform soluble fraction was inactive in the antidiarrhoeal test, the lipophilic limonoids probably do not contribute to the observed biological effect. Recent research has, however, shown a variety of other biological effects for these compounds. Toxicity studies and controlled clinical studies are warranted to make the human use of this herbal medicine safer.

## 3. General Discussion

This review indicates that *D. peregrina*, *I. coccinea*, *P. pinnata*, *R. mucronata*, *X. granatum*, and *X. moluccensis* may be considered as potential sources of antidiarrhoeal phytomedicines or herbal drugs. They show promising potential, since their intake may counteract the diarrhoeal gastrointestinal symptoms in animal models and act against pathogenic microorganisms that cause diarrhoea. For *H. littoralis*, less scientific evidence has been obtained at present, in spite of its documented traditional use in treatment of diarrhoea. However, the scientific literature concerning antidiarrhoeal effects of most plant remedies is quite limited, and on the basis of the available information we are not able to recommend one single species for future exploitation as an antidiarrhoeal drug in this area. Additional investigations are required to elucidate the exact mechanism of action and possible toxic effects. Properly designed clinical trials of promising herbal medicines should also be performed to ascertain the optimal dosages, formulation and effects. A common feature of several of the presented antidiarrhoeal plants is the high content of tannins or proanthocyanidins, which are known from previous studies to have antidiarrhoeal activities [266,267,268]. The efficacy of some flavonoids, e.g., catechins and proanthocyanidins, and proanthocyanidin-rich extracts as antidiarrhoeal agents has been shown in a number of investigations [21,269,270,271]. Since proanthocyanidins usually are very good antioxidants, it is noteworthy that well known inducers of dysentery such as *S. dysenteria* toxin [272] and *Entamoeba histolytica* lectin [273] have been reported to increase oxidative stress in intestinal cells. A similar pro-oxidative effect has been reported for chronic diarrhoea [274]. In addition, ulcerative colitis has been suggested to be related to neutrophil-dependent oxidative stress [275]. It is plausible that phenolic compounds are involved in the medical effects of several of the plants from Bangladesh discussed in this review. In many cases, the bark is the part of the plant traditionally used to treat diarrhoea, and barks are often good sources of tannins. However, the extracts showing positive effects should be investigated further by biological activity-guided searching for active compounds which may lead to concentrated extract with bioactive compounds, excluding toxic, unhealthy and undesirable substances. 

Every year, thousands of people in Bangladesh and millions worldwide are affected by diarrhoeal diseases. Especially children are suffering. For the rural people in Bangladesh it is extremely important to have access to safe and effective medicines to reduce the mortality and morbidity of diarrhoea. In order to reach that goal, development of local resources such as traditional plant medicines may be a good solution. An increased focus on phytomedicines with antidiarrhoeal effects may help relieving the local population from symptoms, disease and death. A local utilization of medicinal plants for the development and production of herbal medicines and/or phytomedicines may also give the people involved work and income, securing their private economy. Protection of intellectual property rights has to be maintained when exploring the plant material in order to produce phytomedicines and herbal drugs for commercial use, e.g., local production, local sale, simple procedure, income to local community. It is important to guarantee that appropriate institutions, local communities or other involved parts get a fair compensation for their knowledge. From an ecological point of view, sustainable uses of the species have to be guaranteed, as well. It is well known that mangrove forests are threatened as ecosystems. All over the world mangrove forests disappear due to deforestation, urbanization, global changes and pollution. Industrialized fishery, shrimp farming and plantations of rice, coconut and palm oil lead to deforestation, and the result may be that the basis for existence of the mangrove species disappears [23,65,70]. 

As previously pointed out, diarrhoea is a major problem among children, and therefore the development of antidiarrhoeal preparations to children should be a research purpose of high priority. However, drug pharmacokinetics is different in children than in adults, differing in the adsorption, distribution, metabolism and excretion of drugs. The children can be even more susceptible to the toxic and adverse effects of plant products than adults, and even if the medicines are safe for adults, one should be careful administrating the same agents to children until safety studies have been performed. Today, limited information is available about the efficacy and safety of herbal medications in children. Because of these factors, there is an increased risk carrying out clinical studies in children compared to adults [276]. 

Natural products may have advantages compared to conventional modern medicines. The complex content of chemicals may have multiple targets of action and might therefore have several potential effects against diarrhoeal diseases. The involvement of synergistic effects is likely too, e.g., astringent and antibacterial effect in combination with decreased intestinal movements. Thus, separating the individual components of a plant extract may lead to loss of activity, since it is often the unique combination of chemical compounds that contribute to the desired effect. Natural products also have a benefit, especially in developing countries, since the costs are much lower compared to modern pharmaceuticals. The majority of the world’s population (60%–70%) relies solely upon medicinal plants as treatment for diseases [277]. One important reason is the high costs of drugs produced by the pharmaceutical industry, but cultural and social factors are also influencing the choice of drug. 

## 4. Conclusions

In conclusion, we have found that some of the plants used as antidiarrhoeals in the Sundarbans contain constituents that make their efficacy in this respect likely. For six of the plants discussed in this paper (*D. peregrina*, *I. coccinea*, *P. pinnata*, *R. mucronata*, *X. granatum* and *X. moluccensis*), pharmacological experiments support this conclusion. Clinical studies and tests for toxicity are largely missing, and such experiments should be carried out in order to have a basis on which plants to recommend as phytomedicines useful against diarrhoea.

## References

[1] Kosek M., Bern C., Guerrant R.L. (2003). The global burden of diarrhoeal disease, as estimated from studies published between 1992 and 2000. Bull. World Health Organ..

[2] World Health Organization (WHO) (2005). The World Health Report 2005—Make Every Mother and Child Count.

[3] World Health Organization (WHO) (2007). The World Health Report 2007—A Safer Future: Global Public Health in the 21st Century.

[4] Lutterodt G.D. (1992). Inhibition of microlax-induced experimental diarrhoea with narcotic-like extracts of *Psidium guajava* leaf in rats. J. Ethnopharmacol..

[5] Albert M.J., Faruque A.S.G., Faruque S.M., Sack R.B., Mahalanabis D. (1999). Case-control study of enteropathogens associated with childhood diarrhea in Dhaka, Bangladesh. J. Clin. Microbiol..

[6] Sack R.B., Siddique A.K., Longini I.M., Nizam A., Yunus M., Islam M.S., Morris J.G., Ali A., Huq A., Nair G.B. (2003). A 4-year study of the epidemiology of *Vibrio cholerae* in four rural areas of Bangladesh. J. Infect. Dis..

[7] Qadri F., Khan A.I., Faruque A.S.G., Begum Y.A., Chowdhury F., Nair G.B., Salam M.A., Sack D.A., Svennerholm A.-M. (2005). Enterotoxigenic *Escherichia coli* and *Vibrio cholerae* diarrhea, Bangladesh, 2004. Emerg. Infect. Dis..

[8] Palombo E.A. (2006). Phytochemicals from traditional medicinal plants used in the treatment of diarrhoea: modes of action and effects on intestinal function. Phytother. Res..

[9] Barrett M. (2004). The Handbook of Clinically Tested Herbal Remedies.

[10] Dillinger T.L., Barriga P., Escarcega S., Jimenez M., Lowe D.S., Grivetti L.E. (2000). Food of the gods: cure for humanity? A cultural history of the medicinal and ritual use of chocolate. J. Nutr..

[11] Alam M.A., Akter R., Subhan N., Rahman M.M., Majumder M.M., Nahar L., Sarker S.D. (2008). Antidiarrhoeal property of the hydroethanolic extract of the flowering tops of *Anthocephalus cadamba*. Braz. J. Pharmacogn..

[12] Bruins M.J., Cermak R., Kiers J.L., van der Meulen J., van Amelsvoort J.M.M., van Klinken B.J.W. (2006). *In vivo* and *in vitro* effects of tea extracts on enterotoxigenic Escherichia coli-induced intestinal fluid loss in animal models. J. Pediat. Gastroenterol. Nutr..

[13] Chen J.-C., Ho T.-Y., Chang Y.-S., Wu S.-L., Hsiang C.-Y. (2006). Anti-diarrheal effect of Galla Chinensis on the *Escherichia coli* heat-labile enterotoxin and ganglioside interaction. J. Ethnopharmacol..

[14] Chen J.-C., Huang L.-J., Wu S.-L., Kuo S.-C., Ho T.-Y., Hsiang C.-Y. (2007). Ginger and its bioactive component inhibit enterotoxigenic *Escherichia coli* heat-labile enterotoxin-induced diarrhea in mice. J. Agric. Food Chem..

[15] Lakhsminarayana M., Shivkumar H., Rimaben P., Bhargava V.K. (2011). Antidiarrhoeal activity of leaf extract of *Moringa oleifera* in experimentally induced diarrhoea in rats. Int. J. Phytomed..

[16] Oi H., Matsuura D., Miyake M., Ueno M., Takai I., Yamamoto T., Kubo M., Moss J., Noda M. (2002). Identification in traditional herbal medications and confirmation by synthesis of factors that inhibit cholera toxin-induced fluid accumulation. Proc. Natl. Acad. Sci. USA.

[17] Rahman T., Rahman K.A., Rajia S., Alamgir M., Khan M.T.H., Choudhuri M.S.K. (2008). Evaluation of antidiarrhoeal activity of cardamom (*Elettaria cardamomum*) on mice models. Orient. Pharm. Exp. Med..

[18] Asif H.M., Usmanghni K., Akram M., Akhtar N., Jabeen Q., Shah S.M.A., Rehman R., Ahmed K., Saeed T. (2010). Herbal treatment of secretory diarrhea. Int. J. Phytomed..

[19] Cai Y., Evans F.J., Roberts M.F., Phillipson J.D., Zenk M.H., Gleba Y.Y. (1991). Biological and chemical investigation of dragon’s blood from *Croton* species of South America. Part 1. Polyphenolic compounds from *Croton lechleri*. Phytochemistry.

[20] Lewis W.H., Elvin-Lewis M.P.F. (2003). Medical Botany: Plants Affecting Human Health.

[21] Fischer H., Machen T.E., Widdicombe J.H., Carlson T.J.S., King S.R., Chow J.W.S., Illek B. (2004). A novel extract SB-300 from the stem bark latex of *Croton lechleri* inhibits CFTR-mediated chloride secretion in human colonic epithelial cells. J. Ethnopharmacol..

[22] Patz J.A., Campbell-Lendrum D., Holloway T., Foley J.A. (2005). Impact of regional climate change on human health. Nature.

[23] Iftekhar M.S., Islam M.R. (2004). Degeneration of Bangladesh’s Sundarbans mangroves: A management issue. Int. Forestry Rev..

[24] Mandal R.N., Das C.S., Naskar K.R. (2010). Dwindling Indian sundarban mangrove: The way out. Sci. Cult..

[25] Upadhyay V.P., Ranjan R., Singh J.S. (2002). Human-mangrove conflicts: The way out. Curr. Sci..

[26] Li M.-Y., Xiao Q., Pan J.-Y., Wu J. (2009). Natural products from semi-mangrove flora: Source, chemistry and bioactivities. Nat. Prod. Rep..

[27] Datta D., Chattopadhyay R.N., Deb S. (2011). Prospective livelihood opportunities from mangroves of the Sunderbans, India. Res. J. Environ. Sci..

[28] Kirtikar K.R., Basu B.D. (1935). Indian Medicinal Plants.

[29] Male Flowers of *Diospyros malabarica* from Ebenaceae. Rarely Seen in Cultivation. http://en.wikipedia.org/wiki/File:Malabar_Ebony.jpg.

[30] Chopra R.N., Nayar S.L., Chopra I.C. (1956). Glossary of Indian Medicinal Plants.

[31] Sinha B.N., Bansal S.K. (2008). A review of phytochemical and biological studies of *Diospyros* species used in folklore medicine of Jharkand. J. Nat. Remedies.

[32] Misra P.S., Misra G., Nigam S.K., Mitra C.R. (1971). Constituents of *Diospyros peregrina* fruit and seed. Phytochemistry.

[33] Chinsembu K.C., Hedimbi M. (2010). An ethnobotanical survey of plants used to manage HIV/AIDS opportunistic infections in Katima Mulilo, Caprivi region, Namibia. J. Ethnobiol. Etnomed..

[34] Kantamreddi V.S.S., Wright C.V. (2008). Investigation of *Diospyros* species for antiplasmodial properties. Evid. Based Complement. Altern. Med..

[35] Wagh V.V., Jain A.K., Kadel C. (2011). Ethnomedicinal plants used for curing dysentery and diarrhea by tribals of Jhabua district (Madhya Pradesh). Indian J. Nat. Prod. Resour..

[36] Gupta R.K., Tiwari R.D. (1964). Chemical examination of the bark of *Diospyros peregrina*. Proc. Indian Nat. Sci. Acad. Sect. A.

[37] Sundararamaiah T., Ramraj S.K., Rao K.L., Vimalabai V. (1976). Isolation of the lupeol group of triterpenes from *Dillenia indica* Linn. and *Diospyros peregrina*. J. Indian Chem. Soc..

[38] Chauhan J.S., Kumari G. (1978). A new leucoanthocyanin from the stem of *Diospyros peregrina*. J. Indian Chem. Soc..

[39] Chauhan J.S., Kumari G. (1980). Nonadecan-7-ol-2-one, an aliphatic ketol from *Diospyros peregrina*. Phytochemistry.

[40] Gupta R.K., Tiwari R.D. (1964). Chemical examination of the leaves of *Diospyros peregrina* Gurke. Indian J. Chem..

[41] Jain N., Alam M.S., Kamil M., Ilyas M., Ali M. (1992). Peregrinol: A new lupene type triterpene from *Diospyros peregrina*. Pharmazie.

[42] Bhaumik T., Dey A.K., Das P.C., Mukhopadhyay A.K., Chatterjee M.A. (1981). Triterpenes of *Diospyros peregrina* Gurke: Partial syntheses of olean-9(11),12-dien-3-one and ursan-9(11),12-dien-3-one (marsformosanone). Indian J. Chem. Sect. B Org. Med. Chem..

[43] Pareek R.B., Vidyapati T.J., Bhutani K.K. (2008). Chemical examination of the fruits of *Diospyros peregrina*. J. Teaching Res. Chem..

[44] Jain N., Yadava R. (1996). A novel chromenoflavone from the fruits of *Diospyros peregrina*. Fitoterapia.

[45] Sahu R., Dewanjee S., Dua T.K., Gangopadhyay M., Das A.K., Dey S.P. (2012). Dereplication coupled with *in vitro* antioxidant assay of two flavonoid glycosides from *Diospyros peregrina* fruit. Nat. Prod. Res..

[46] Sampurna T., Rao T.S.S. (2004). Chemical examination of the fruits of *Diospyros peregrina* Gurke. Asian J. Chem..

[47] Begum S.A., Hosain M.M., Ahmed M.F., Muttakin M.A., Rahman M.M. (2009). Vitamin C content in tropical fruits and vegetables available in different districts of Bangladesh. J. Bangladesh Acad. Sci..

[48] Chauhan J.S., Saraswat M., Kumari G. (1979). Structure of a new dihydroflavonol glycoside from *Diospyros peregrina* roots. Planta Med..

[49] Chauhan J.S., Saraswat M., Kumari G. (1982). Structure of a new flavanone glycoside from *Diospyros peregrina* roots. Indian J. Chem. Sect. B Org. Med. Chem..

[50] Rouf R., Uddin S.J., Shilpi J.A., Toufiq-ur-rahman M., Ferdous M.M., Sarker S.D. (2006). Anti-diarrhoeal properties of *Diospyros peregrina* in the castor oil-induced diarrhoea model in mice. Ars. Pharm..

[51] Dewanjee S., Maiti A., Kundu M., Mandal S.C. (2007). Evaluation of anthelmintic activity of extracts of *Diospyros peregrina*, *Coccinia grandis* and *Schima wallichii*. Dhaka Univ. J. Pharm. Sci..

[52] Dewanjee S., Kundu M., Maiti A., Majumdar R., Majumdar A., Mandal S.C. (2007). *In vitro* evaluation of antimicrobial activity of crude extract from plants *Diospyros peregrina*, *Coccinia grandis* and *Swietenia macrophylla*. Trop. J. Pharm. Res..

[53] Dewanjee S., Maiti A., Mandal S.C. (2007). Antioxidant activity of the methanol extract of *Diospyros peregrina* fruit in alloxan induced diabetic rats. Pharmacologyonline.

[54] Dewanjee S., Bose S.K., Sahu R., Mandal S.C. (2008). Antidiabetic effect of matured fruits of *Diospyros peregrina* in alloxan-induced diabetic rats. Int. J. Green. Pharm..

[55] Dewanjee S., Sahu R., Mandal V., Maiti A., Mandal S.C. (2009). Antidiabetic and antioxidant activity of the methanol extract of *Diospyros peregrina* fruit on type I diabetic rats. Pharm. Biol..

[56] Dewanjee S., Gangopadhyay M., Das A.K. (2011). Multimodal approaches of the hydroalcoholic extract of fruits in diabetic therapy. Nat. Prod. Res..

[57] Dewanjee S., Maiti A., Sahu R., Dua T.K., Mandal V. (2011). Effective control of type 2 diabetes through antioxidant defense by edible fruits of *Diospyros peregrina*. Evid. Based Complement. Altern. Med..

[58] Kumar K.E., Mastan S.K., Sreekanth N., Chaitanya G., Sumalatha G., Krishna P.V. (2008). Hypoglycemic and antihyperglycemic activity of aqueous extract of *Diospyros peregrina* fruits in normal and alloxan-induced diabetic rabbits. Pharmacologyonline.

[59] Venu Gopal Y., Ravindranath A., Kalpana G., Raju A.B., Reddy P. (2011). Antitumor activity of *Diospyros peregrina* on Ehrlich ascites carcinoma in mice. J. Sci. Res..

[60] Hussan Reza K., Jeevanandham S., Kumervelrajen R. (2012). Evaluation of *Diospyros peregrina* gum as a novel binder in tablet formulation. Int. Res. J. Pharm. Appl. Sci..

[61] Singh N., Nath R., Gupta M.L. (1988). A pharmacological evaluation of anti-stress activity of *Diospyros peregrina* Gurke. Indian J. Pharm..

[62] Tomlinson P.B. (1986). The Botany of Mangroves.

[63] *Heritiera littoralis* (Green Fruit). Location: Oahu, Keehi Lagoon. http://en.wikipedia.org/wiki/File:Starr_080530-4634_Heritiera_littoralis.jpg.

[64] Daengrot C., Ponglimanont C., Karalai C. Chemical Constituents from the Barks of Heritiera Littoralis. Proceedings of the 31st Annual Congress on Science and Technology of Thailand.

[65] Bandaranayake W.M. (1998). Traditional and medicinal uses of mangroves. Mangroves Salt Marshes.

[66] Bandaranayake W.M. (2002). Bioactivities, bioactive compounds and chemical constitutions of mangrove plants. Wetlands Ecol. Manag..

[67] Pattanaik C., Reddy C.S., Dhal N.K., Das R. (2008). Utilisation of mangrove forests in Bhitarkanika wildlife sanctuary, Orissa. Indian J. Tradit. Knowl..

[68] Miles D.H., Lho D.S., de la Cruz A.A., Gomez E.D., Weeks J.A., Atwood J.L. (1987). Toxicants from mangrove plants. 3. Heritol, a novel ichthyotoxin from the mangrove plant *Heritiera littoralis*. J. Org. Chem..

[69] Bhargava N. (1983). Ethnobotanical studies of the tribes of Andaman and Nicobar Islands, India. I. Onge. Econ. Bot..

[70] Dahdouh-Guebas F., Collin S., lo Seen D., Rönnbäck P., Depommier D., Ravishankar T., Koedam N. (2006). Analysing ethnobotanical and fishery-related importance of mangroves of the East-Godavari Delta (Andhra Pradesh, India) for conservation and management purposes. J. Ethnobiol. Ethnomed..

[71] Tewtrakul S., Tansakul P., Daengrot C., Ponglimanont C., Karalai C. (2010). Anti-inflammatory principles from *Heritiera littoralis* bark. Phytomedicine.

[72] Balasooriya S.J., Sotheeswaran S. (1982). Economically useful plants of Sri Lanka. Part IV. Screening of Sri Lanka plants for tannins. J. Natl. Sci. Counc. Sri Lanka.

[73] Tian Y., Wu J., Zhang S. (2004). Flavonoids from leaves of *Heritiera littoralis* D. J. Chin. Pharm. Sci..

[74] Takeda Y., Miyazaki K., Shimizu H., Masuda T., Hirata E., Takushi A., Shinzato T., Otsuka H. (2000). A new phenylpropanoid-glycerol conjugate from *Heritiera littoralis* Dryand. Nat. Med..

[75] Gaydou E.M., Ramanoelina A.R.P., Rasoarahona J.R.E., Combres A. (1993). Fatty acid composition of *Sterculia* seeds and oils from Madagascar. J. Agric. Food Chem..

[76] Miles D.H., Chittawong V., Lho D.-S., Payne A.M., de la Cruz A.A., Gomez E.D., Weeks J.A., Atwood J.L. (1991). Toxicants from mangrove plants, VII. Vallapin and vallapianin, novel sesquiterpene lactones from the mangrove plant *Heritiera littoralis*. J. Nat. Prod..

[77] Miles D.H., de la Cruz A.A., Ly A.-M., Lho D.S., Gomez E.D., Weeks J.A., Atwood J.L. (1987). Toxicants from mangrove plants. Ichthyotoxins from the Philippine plant *Heritiera littoralis*. Am. Chem. Soc. Symp. Ser..

[78] Miles D.H., Ly A.-M., Chittawong V., de la Cruz A.A., Gomez E.D. (1989). Toxicants from mangrove plants, VI. Heritonin, a new piscicide from the mangrove plant *Heritiera littoralis*. J. Nat. Prod..

[79] Dang H.C.T. (2007). Antioxidants in *Heritiera fomes*, a Medicinal Plant from the Mangrove Forest of Bangladesh (in Norwegian). Master Thesis.

[80] Tao W.Q., Xu M.B., Huang L.Y., Miao S.Y. (2012). Antibacterial activity of extracts from four mangrove species *in vitro*. Med. Plant.

[81] Latha P.G., Panikkar K.R., Suja S.R., Abraham A., Rajasekharan S. (2001). Chemistry, pharmacognosy, pharmacology and botany of *Ixora coccinea*—A review. J. Med. Arom. Plant Sci..

[82] A Specimen of *Ixora coccinea*, Also Known as the Jungle Flame, Shot in Miami, Florida. http://en.wikipedia.org/wiki/File:IxoraCoccineaMiami.JPG.

[83] Nayak S., Udupa L., Udupa S. (2003). Altered antioxidant enzyme profile in wound healing. Indian J. Clin. Biochem..

[84] Ragasa C.Y., Tiu F., Rideout J.A. (2004). New cycloartenol esters from *Ixora coccinea*. Nat. Prod. Res..

[85] Ratnasooriya W.D., Deraniyagala S.A., Bathige S.D.N.K., Goonasekara C.L., Jayakody J.R.A.C. (2005). Antinociceptive action of aqueous extract of the leaves of *Ixora coccinea*. Acta Biol. Hung..

[86] Khare C.P. (2007). Indian Medicinal Plants: An Illustrated Dictionary.

[87] Alam M.K. (1992). Medical ethnobotany of the Marma tribe of Bangladesh. Econ. Bot..

[88] Baliga M.S., Kurian P.J. (2012). *Ixora coccinea* Linn.: Traditional uses, phytochemistry and pharmacology. Chin. J. Integr. Med..

[89] Zachariah R., Nair C.R.S., Panicker P.V. (1994). Anti-inflammatory and anti-mitotic activities of lupeol isolated from the leaves of *Ixora coccinea* Linn. Indian J. Pharm. Sci..

[90] Idowu T.O., Ogundaini A.O., Salau A.O., Obuotor E.M., Bezabih M., Abegaz B.M. (2010). Doubly linked, A-type proanthocyanidins trimer and other constituents of *Ixora coccinea* leaves and their antioxidant and antibacterial properties. Phytochemistry.

[91] Sumathy H., Sangeetha J., Vijayalakhsmi K. (2011). Chromatographic fingerprint analysis of *Ixora coccinea* methanolic flower extract. Int. J. Pharm. Sci. Drug Res..

[92] Lee C.-L., Liao Y.-C., Hwang T.-L., Wu C.-C., Chang F.-R., Wu Y.-C. (2010). Ixorapeptide I and ixorapeptide II, bioactive peptides isolated from *Ixora coccinea*. Bioorg. Med. Chem. Lett..

[93] Yadava R.N. (1989). Analysis of the fixed oil from the roots of *Ixora coccinea* Linn. Asian J. Chem..

[94] Srinivasan G.V., Sharanappa P., Udayan P.S., Leela N.K., Vijayan K.K. (2010). Chemical composition and antimicrobial activity of the essential oil of *Ixora coccinea* L. root. J. Med. Arom. Plant Sci..

[95] Maniyar Y., Bhixavatimath P., Agashikar N.V. (2010). Antidiarrheal activity of *Ixora coccinea* Linn. in rats. J. Ayurveda Integr. Med..

[96] Annapurna J., Amarnath P.V.S., Amar Kumar D., Ramakrishna S.V., Raghavan K.V. (2003). Antimicrobial activity of *Ixora coccinea* leaves. Fitoterapia.

[97] Sasidharan V.K. (1997). Search for antibacterial and antifungal activity of some plants of Kerala. Acta Pharm..

[98] Selvaraj N., Lakshmanan B., Mazumder P.M., Karuppusamy M., Jena S.S., Pattnaik A.K. (2011). Evaluation of wound healing and antimicrobial potentials of *Ixora coccinea* root extract. Asia Pac. J. Trop. Med..

[99] Shyamal S., Latha P.G., Suja S.R., Shine V.J., Anuja G.I., Sini S., Pradeep S., Shikha P., Rajasekharan S. (2010). Hepatoprotective effect of three herbal extracts on aflatoxin B1-intoxicated rat liver. Singap. Med. J..

[100] Torey A., Sasidharan S., Latha L.Y., Sudhakaran S., Ramanathan S. (2010). Antioxidant activity and total phenolic content of methanol extracts of *Ixora coccinea*. Pharm. Biol..

[101] Momin F.N., Kalai B.R., Shikalgar T.S., Naikwade N.S. (2012). Cardioprotective effect of methanolic extract of *Ixora coccinea* Linn. leaves on doxorubicin-induced cardiac toxicity in rats. Indian J. Pharmacol..

[102] Haridass S., Sekar S., Vijayan R., Jayakumar S., Thtmizharasu S., Krishnamurthy V., Chidambaram S.B. (2012). Relative antioxidant and cytotoxic activities of *Ixora coccinea* flower extracts. J. Pharm. Res..

[103] Wongwattanasathien O., Kangsadalampai K., Tongyonk L. (2010). Antimutagenicity of some flowers grown in Thailand. Food Chem. Toxicol..

[104] Maniyar Y., Bhixavatimath P. (2011). Evaluation of the hypoglycaemic and hypolipidaemic activities of the aqueous extract of the leaves of *Ixora coccinea* Linn in diabetic rats. J. Clin. Diagnost. Res..

[105] Bhattacharya A., Kar D.R., Sengupta A., Ghosh G., Mishra S.K. (2011). Evaluation of anti-inflammatory and analgesic activity of *Ixora coccinea* flower extract. Asian J. Chem..

[106] Kensa V.M. (2011). Larvicidal effects of selected plant leaves extract against *Anopheles* mosquitoes. Ecol. Environ. Conserv. Paper.

[107] Seetha D.B., Sudhakaran-Nair C.R., Velayudha-Panicker P. (1991). Pharmacognostical and pharmacological studies on the root of *Ixora coccinea* Linn. (Rubicaceae). Indian J. Pharm. Sci..

[108] Latha P.G., Panikkar K.R. (2000). Inhibition of chemical carcinogenesis in mice by *Ixora coccinea* flowers. Pharm. Biol..

[109] Atiq-Ur-Rahman, Taqvi S.I.H., Versiani M.A., Ikram A., Ahmed S.K. (2012). Effects of whole flower and fractions from *Ixora coccinea* Linn. on cardiovascular system: A preliminary report. J. Chem. Soc. Pak..

[110] Howell A.B., Reed J.D., Krueger C.G., Winterbottom R., Cunningham D.G., Leahy M. (2005). A-type cranberry proanthocyanidins and uropathogenic bacterial anti-adhesion activity. Phytochemistry.

[111] Chopade V.V., Tankar A.N., Pande V.V., Tekade A.R., Gowekar N.M., Bhandari S.R., Khandake S.N. (2008). *Pongamia pinnata*: Phytochemical constituents, traditional uses and pharmacological properties: A review. Int. J. Green Pharm..

[112] Pandey A., Khatri P., Patel P., Jakhetia V., Sharma S. (2009). Phytopharmacognostic and pharmacological review of *Pongamia pinnata* family Fabaceae. J. Global Pharm. Technol..

[113] Satish Kumar B.N. (2011). Phytochemistry and pharmacological studies of *Pongamia pinnata* (Linn.) Pierre. Int. J. Pharm. Sci. Rev. Res..

[114] Khatri P., Patel R. (2013). A phytochemical overview of various parts of *Pongamia pinnata* (Karanj). World J. Pharm. Res..

[115] *Pongamia pinnata*—Karanj, Indian Beech Tree, Honge Tree, Pongam Tree or Panigrahi at Deer Park in Shamirpet, Rangareddy District, Andhra Pradesh, India. http://en.wikipedia.org/wiki/File:Pongamia_pinnata_%28Karanj%29_near_Hyderabad_W_IMG_7633.jpg.

[116] Muthu C., Ayyanar M., Raja N., Ignacimuthu S. (2006). Medicinal plants used by traditional healers in Kancheepuram district of Tamil Nadu, India. J. Ethnobiol. Ethnomed..

[117] Pandikumar P., Chellappandian M., Mutheeswaran S., Ignacimuthu S. (2011). Consensus of local knowledge on medicinal plants amongst traditional healers in Mayialadumparai block of Theni district, Tamil Nadu, India. J. Ethnopharmacol..

[118] Ragupathy S., Newmaster S.G. (2009). Valorizing the “Irulas” traditional knowledge of medicinal plants in the Kodiakkal Reserve Forest, India. J. Ethnobiol. Ethnomed..

[119] Awashti A.K. (1991). Ethnobotanical studies of the Negrito islanders of Andaman islands, India—The great Andamanese. Econ. Bot..

[120] Kumar A., Pandey V.C., Tewari D.D. (2012). Documentation and determination of consensus about phytotherapeutic veterinary practices among the Tharu tribal community of Uttar Pradesh, India. Trop. Animal Health Prod..

[121] Jain S.K. (1965). Medicinal plant lore of the tribals of Bastar. Econ. Bot..

[122] Garg G.P. (1979). A new component from leaves of *Pongamia glabra*. Planta Med..

[123] Mahey S., Sharma P., Seshadri T.R., Mukerjee T.R. (1972). Structure & synthesis of glabrachromene, a new constituent of *Pongamia glabra*. Indian J. Chem..

[124] Malik S.B., Seshadri T.R., Sharma P. (1976). Minor components of the leaves of *Pongamia glabra*. Indian J. Chem..

[125] Malik S.B., Sharma P., Seshadri T.R. (1977). Furanoflavonoids from the leaves of *Pongamia glabra*. Indian J. Chem..

[126] Sagwan S., Rao D.V., Sharma R.A. (2012). Studies on GC/MS spectroscopic analysis of some different *in vivo* plant extracts of (Karanj) *Pongamia pinnata* (L.) Pierre. J. Global Pharma Technol..

[127] Talapatra B., Mallik A.K., Talapatra S.K. (1985). Triterpenoids and flavonoids from the leaves of *Pongamia glabra* Vent. Demethylation studies on 5-methoxy-furanoflavones. J. Indian Chem. Soc..

[128] Shoba F.G., Thomas M. (2001). Study of antidiarrhoeal activity of four medicinal plants in castor-oil induced diarrhoea. J. Ethnopharmacol..

[129] Brijesh S., Daswani P.G., Tetali P., Rojatkar S.R., Antia N.H., Birdi T.J. (2006). Studies on *Pongamia pinnata* (L.) Pierre leaves: Understanding the mechanism(s) of action in infectious diarrhea. J. Zhejiang Univ. Sci. B.

[130] Dahikar S.B., Arote S.R., Tambekar D.H., Yeole P.G., Bhutada S.A. (2009). Antibacterial properties of *Pongamia pinnata* Linn. (Fabaceae) against enteric bacterial pathogens. Biotechnol. Indian J..

[131] Johnson D.B., Shringi B.N., Patidar D.K., Chalichem N.S.S., Javvadi A.K. (2011). Screening of antimicrobial activity of alcoholic & aqueous extracts of some indigenous plants. Indo-Global J. Pharm. Sci..

[132] Ramesh K., Gifson Gnanadhas Y., Karthick N., Umamaheswari S. (2011). Plasmid mediated drug resistance and anti-MRSA activity of *Pongamia glabra* and *Ocimum basilicum*. J. Pharm. Res..

[133] Sumathi R., Pavni S., Sivakumar T. (2009). Antimicrobial evaluation of lipid extract of *Pongamia pinnata* leaves. Res. J. Pharm. Technol..

[134] Sajid Z.I., Anwar F., Shabir G., Rasul G., Alkharfy K.M., Gilani A.-H. (2012). Antioxidant, antimicrobial properties and phenolics of different solvent extracts from bark, leaves and seeds of *Pongamia pinnata* (L.) Pierre. Molecules.

[135] Sharma A., Tyagi S., Nag R., Chaturvedi A., Nag T.N. (2011). Antimicrobial activity and cellular toxicity of flavonoid extracts from *Pongamia pinnata* and *Vitex negundo*. Rom. Biotechnol. Lett..

[136] Umera Begam A.K., Manoharan N., Sirajudeen J., Abdul Jameel A. (2010). Effect of some Indian traditional plants on few common pathogens. Adv. Appl. Sci. Res..

[137] Gupta V., Sharma M. (2011). *In vitro* antioxidant studies on methanolic extracts of *Pongamia pinnata* (L.) Pierre: A great versatile leguminous plant. J. Pharm. Res..

[138] Badole S.L., Bodhankar S.L. (2009). Antihyperglycemic activity of cycloart-23-ene-3β, 25-diol isolated from stem bark of *Pongamia pinnata* in alloxan induced diabetic mice. Res. J. Phytochem..

[139] Badole S.L., Bodhankar S.L. (2009). vestigation of antihyperglycaemic activity of aqueous and petroleum ether extract of stem bark of *Pongamia pinnata* on serum glucose level in diabetic mice. J. Ethnopharmacol..

[140] Badole S.L., Bodhankar S.L. (2010). Antidiabetic activity of cycloart-23-ene-3β, 25-diol isolated from *Pongamia pinnata* (L. Pierre) in streptozocine-nicotinamide induced diabetic mice. Eur. J. Pharmacol..

[141] Sikarwar M.S., Patil M.B. (2010). Antidiabetic activity of *Pongamia pinnata* leaf extracts in alloxan-induced diabetic rats. Int. J. Ayurveda Res..

[142] Badole S.L., Zanwar A.A., Khopade A.N., Bodhankar S.L. (2011). *In vitro* antioxidant and antimicrobial activity cycloart-23-ene-3β, 25-diol (B2) isolated from *Pongamia pinnata* (L. Pierre). Asia Pac. J. Trop. Med..

[143] Jaiswal N., Yadav P.P., Maurya R., Srivastava A.K., Tamrakar A.K. (2011). Karanjin from *Pongamia pinnata* induces GLUT4 translocation in skeletal muscle cells in a phosphatidylinositol-3-kinase-independent manner. Eur. J. Pharmacol..

[144] Tamrakar A.K., Jaiswal N., Yadav P.P., Maurya R., Srivastava A.K. (2011). Pongamol from *Pongamia pinnata* stimulates glucose uptake by increasing surface GLUT4 level in skeletal muscle. Mol. Cell. Endocrinol..

[145] Tamrakar A.K., Yadav P.P., Tiwari P., Maurya R., Srivastava A.K. (2008). Identification of pongamol and karanjin as lead compounds with antihyperglycemic activity from *Pongamia pinnata* fruits. J. Ethnopharmacol..

[146] Prabha T., Dorababu M., Goel S., Agarwal P.K., Singh A., Joshi V.K., Goel R.K. (2009). Effect of methanolic extract of *Pongamia pinnata* Linn seed on gastro-duodenal ulceration and mucosal offensive and defensive factors in rats. Indian J. Exp. Biol..

[147] Vismaya, Belagihally S.M., Rajashekhar S., Jayaram V.B., Dharmesh S.M., Thirumakudalu S.K.C. (2011). Gastroprotective properties of karanjin from Karanja (*Pongamia pinnata*) seeds; Role as antioxidant and H^+^, K^+^-ATPase inhibitor. Evid. Based Complement. Altern. Med..

[148] Bhatia G., Puri A., Maurya R., Yadav P.P., Khan M.M., Khanna A.K., Saxena J.K. (2008). Anti-dyslipidemic and antioxidant activities of different fractions of *Pongamia pinnata* (Lin.) fruits. Med. Chem. Res..

[149] Sagar S.K., Sehgal S.S. (1996). Effects of aqueous extract of deoiled neem (Azadirachta indica A. Juss) seed kernel and karanja (*Pongamia glabra* Vent) seed kernel against *Culex quinquefasciatus*. J. Commun. Dis..

[150] Sagar S.K., Sehgal S.S., Agarwala S.P. (1999). Bioactivity of ethanol extract of Karanja (*Pongamia glabra* Vent) seed coat against mosquitoes. J. Commun. Dis..

[151] Abdul Rahuman A., Bagavan A., Kamaraj C., Vadivelu M., Zahir A.A., Elango G., Pandiyan G. (2009). Evaluation of indigenous plant extracts against larvae of *Culex quinquefasciatus* Say (Diptera: Culicidae). Parasitol. Res..

[152] Swathi S., Murugananthan G., Ghosh S.K. (2010). Oviposition deterrent activity from the ethanolic extract of *Pongamia pinnata*, *Coleus forskohlii*, and *Datura stramonium* leaves against *Aedes aegypti* and *Culex quinquefasciatus*. Pharmacogn. Mag..

[153] Samuel A.J.S.J., Radhamani S., Gopinath R., Kalusalingam A., Vimala A.G.K.A., Husain H.A. (2009). *In vitro* screening of anti-lice activity of *Pongamia pinnata* leaves. Korean J. Parasitol..

[154] Shanthi S., Elamathy S., Panneerselvam A., Radha N. (2011). Antixanthomonas activity of *Pongamia pinnata* Linn. leaves—Cow urine extract—Natural cost effective ecofriendly remedy to bacterial leaf blight of paddy (BLB). J. Pharm. Res..

[155] Srinivasan K., Muruganandan S., Lal J., Chandra S., Tandan S.K., Prakash V.R. (2001). Evaluation of anti-inflammatory activity of *Pongamia pinnata* leaves in rats. J. Ethnopharmacol..

[156] Baki M.A., Khan A., Al-Bari M.A.A., Mosaddik A., Sadik G., Mondal K.A.M.S.H. (2007). Sub-acute toxicological studies of pongamol isolated from *Pongamia pinnata*. Res. J. Med. Med. Sci..

[157] *Rhizophora mucronata* Propagules at Iriomote is. Okinawa, Japan. http://en.wikipedia.org/wiki/File:Rhizophora_mucronata_Propagules.jpg.

[158] Patra J.K., Thatoi H.N. (2011). Metabolic diversity and bioactivity screening of mangrove plants: A review. Acta Physiol. Plant..

[159] Nurulhuda M.N., Chew L.T., Mohd N.M.Y., Abdul R.M.A. (1990). Tannin properties of *Rhizophora* mucronata barks of different ages. Holz Roh Werkst..

[160] Shinoda Y., Ogisu M., Iwata S., Tajima T. (1985). The chemical composition of mangroves II. Gifu Daigaku Nogakubu Kenkyu Hokoku.

[161] Rohini R.M., Das A.K. (2010). Antidiarrheal and anti-inflammatory activities of lupeol, quercetin, β-sitosterol, adene-5-3-ol and caffeic acid isolated from *Rhizophora mucronata* bark. Pharm. Lett..

[162] Rohini R.M., Das A.K. (2010). Triterpenoids from the stem bark of *Rhizophora mucronata*. Nat. Prod. Res..

[163] Rohini R.M., Das A.K. (2011). Determination of lupeol, β-sitosterol and quercetin from ethyl acetate extract of *Rhizophora mucronata* bark by HPTLC technique. Asian J. Pharm. Clin. Res..

[164] Saha P.K., Ganguly T., Ganguly S.N., Sircar S.M. (1978). Rhizophorine, a new indole acid plant growth inhibitor from *Rhizophora mucronata*. Plant Biochem. J..

[165] Ganguly S.N., Sircar S.M. (1974). Gibberellins from mangrove plants. Phytochemistry.

[166] Joel E.L., Bhimba V. (2010). Isolation and characterization of secondary metabolites from the mangrove plant *Rhizophora mucronata*. Asia Pac. J. Trop. Med..

[167] Hogg R.W., Gillan F.T. (1984). Fatty acids, sterols and hydrocarbons in the leaves from eleven species of mangrove. Phytochemistry.

[168] Chandrasekaran M., Kumar A.S., Kannathasan K., Venkatesalu V. (2010). Fatty-acid composition of some mangroves. Chem. Nat. Comp..

[169] Lakshmi V., Misra A. (1995). The novel 1-hydroxy-5-oxobicyclo[6.4.0]dodecane from *Rhizophora mucronata*. Planta Med..

[170] Puspitasar Y.E., Hartiati A.M., Suprayitno E. (2012). The potency of *Rhizophora mucronata* leaf extract as antidiarrhea. J. Appl. Sci. Res..

[171] Laphookhieo S., Karalai C., Ponglimanont C. (2004). New sesquiterpenoid and triterpenoids from the fruits of *Rhizophora mucronata*. Chem. Pharm. Bull..

[172] Anjaneyulu A.S.R., Anjaneyulu V., Rao V.L. (2002). New beyerane and isopimarane diterpenoids from *Rhizophora mucronata*. J. Asian Nat. Prod. Res..

[173] Anjaneyulu A.S.R., Anjaneyulu V., Rao V.L. (2000). Rhizophorin B: A novel beyerane diterpenoid from the Indian mangrove plant *Rhizophora mucronata*. Indian J. Chem. Sect. B.

[174] Anjaneyulu A.S.R., Rao V.L. (2001). Rhizophorin A, a novel secolabdane diterpenoid from the Indian mangrove plant *Rhizophora mucronata*. Nat. Prod. Lett..

[175] Richter A., Thonke B., Popp M. (1990). 1d-1-*O*-Methyl-muco-inositol in *Viscum album* and members of the Rhizophoraceae. Phytochemistry.

[176] Ahmed S.S., Hiader S.I., Rabbani M.M. (1988). 25-*S*-Spirost-5-ene-3β, 14α-diol from *Rhizophora mucronata*. Fitoterapia.

[177] Premanathan M., Kathiresan K., Chandra K. (1995). Antiviral evaluation of some marine plants against Semliki forest virus. Pharm. Biol..

[178] Premanathan M., Kathiresan K., Yamamoto N., Nakashima H. (1999). *In vitro* anti-human immunodeficiency virus activity of polysaccharide from *Rhizophora mucronata* Poir. Biosci. Biotechnol. Biochem..

[179] Premnathan M., Chandra K., Bajpai S.K., Kathiresan K. (1992). A survey of some Indian marine plants for antiviral activity. Bot. Mar..

[180] Baskaran R., Mohan P.M. (2012). *In vitro* antibacterial activity of leaf extracts of *Rhizophora mucronata* L. against multi drug resistant *Vibrio* spp. isolated from marine water lobster’s larvae hatcheries. Indian J. Geo-Mar. Sci..

[181] Chandrasekaran M., Venkatesalu V., Sivasankari S., Kannathasan K., Sajit Khan A.K., Prabhakar K., Rajendran S., Lakhsmi Sarayu Y. (2006). Antibacterial activity of certain mangroves against methicillin resistant *Staphylococcus aureus*. Seaweed Res. Util..

[182] Rajaretinam R.K., PrakashVincent S.G. (2010). Isolation of a novel bioactive compound from *Rhizophora mucronata* for methicillin resistant *Staphylococcus aureus* (MRSA) and compound toxicity assessment in zebra fish embryos. J. Pharm. Res..

[183] Kusuma S., Anil Kumar P., Boopalan K. (2011). Potent antimicrobial activity of *Rhizophora mucronata*. J. Ecobiotechnol..

[184] Ravikumar S., Inbaneson S.J., Suganthi P., Guanadesigan M. (2011). *In vitro* antiplasmodial activity of ethanolic extracts of mangrove plants from South East coast of India against chloroquine-sensitive *Plasmodium falciparum*. Parasitol. Res..

[185] Ravikumar S., Inbaneson S.J., Suganthi P., Venkatesan M., Ramu A. (2011). Mangrove plants as a source of lead compounds for the development of new antiplasmodial drugs from South East coast of India. Parasitol. Res..

[186] Agoramoorthy G., Chen F.A., Venkatesalu V., Kuo D.H., Shea P.C. (2008). Evaluation of antioxidant polyphenols from selected mangrove plants of India. Asian J. Chem..

[187] Vadlapudi V., Naidu K.C. (2009). Evaluation of antioxidant potential of selected mangrove plants. J. Pharm. Res..

[188] Johnson T. (1999). CRC Ethnobotany Desk Reference.

[189] Cannonball Mangrove (*Xylocarpus granatum*) in a Mangrove Swamp in the Daintree Region of Queensland, Australia. Located on the Mardjja Botanical Walk (Coordinates Imprecise). http://en.wikipedia.org/wiki/File:Xylocarpus_granatum.jpg.

[190] Alvi K.A., Crews P., Aalbersberg B., Prasad R. (1991). Limonoids from the Fijian medicinal plant dabi (*Xylocarpus*). Tetrahedron.

[191] Weiner M.A. (1971). Ethnomedicine in Tonga. Econ. Bot..

[192] Rouf R., Uddin S.J., Shilpi J.A., Alamgir M. (2007). Assessment of antidiarrhoeal activity of the methanol extract of *Xylocarpus granatum* bark in mice model. J. Ethnopharmacol..

[193] Uddin S.J., Shilpi J.A., Delazar A., Nahar L., Sarker S.D. (2004). Free radical scavenging activity of some Bangladeshi plant extracts. Orient. Pharm. Exp. Med..

[194] Wiart C. (2006). Medicinal Plants of Asia and the Pacific.

[195] Lakhsmi V., Saxena A., Singh S., Pal R., Srivastava S., Srivastava M.N. (2006). A Process for the Isolation of Standardized Antidiarrhoeal Fraction and Its Active Compounds from the Fruit Seed Coat of *Xylocarpus granatum* Koen and Its Use Thereof. Indian Pat..

[196] Zaridah M.Z., Idid S.Z., Wan Omar A., Khozirah S. (2001). *In vitro* antifilarial effects of three plant species against adult worms of subperiodic *Brugia malayi*. J. Ethnopharmacol..

[197] Wangensteen H., Alamgir M., Duong G.M., Grønhaug T.E., Samuelsen A.B., Malterud K.E., Eddouks M. (2009). Chemical and Biological Studies of Medicinal Plants from the Sundarbans Mangrove Forest. Advances in Phytotherapy Research.

[198] Wangensteen H., Duong G.M., Alamgir M., Sarder M., Samuelsen A.B., Malterud K.E. (2006). Biological activities of limonoids, catechins, procyanidins and extracts from *Xylocarpus granatum*. Nat. Prod. Commun..

[199] Cui J., Deng Z., Li J., Fu H., Proksch P., Lin W. (2005). Phragmalin-type limonoids from the mangrove plant *Xylocarpus granatum*. Phytochemistry.

[200] Wu J., Xiao Q., Huang J.S., Xiao Z.H., Qi S.H., Li Q.X., Zhang S. (2004). Xyloccensins O and P, unique 8,9,30-phragmalin *ortho* esters from *Xylocarpus granatum*. Org. Lett..

[201] Wu J., Xiao Q., Zhang S., Li X., Xiao Z.H., Ding H.X., Li Q.X. (2005). Xyloccensins Q-V, six new 8,9,30-phragmalin *ortho* ester antifeedants from the Chinese mangrove *Xylocarpus granatum*. Tetrahedron.

[202] Wu J., Zhang S., Xiao Q., Li Q., Huang J., Xiao Z., Long L. (2003). Xyloccensin M and N, two new B, D-*seco* limonoids from *Xylocarpus granatum*. Z. Naturforsch. Sect. B.

[203] Wu J., Zhang S., Xiao Q., Li Q.X., Huang H.S., Long L.J., Huang L.M. (2004). Xyloccensin L, a novel limonoid from *Xylocarpus granatum*. Tetrahedron Lett..

[204] Sundarasivarao B., Nazma, Madhusudhanarao J. (1977). Antifungal activity of gedunin. Curr. Sci..

[205] Du S., Wang M., Zhu W., Qin Z. (2009). A new fungicidal lactone from *Xylocarpus granatum* (Meliaceae). Nat. Prod. Res..

[206] Hu W.-M., Wu J. (2010). Protoxylogranatin B, a key biosynthetic intermediate from *Xylocarpus granatum*: Suggesting an oxidative cleavage biogenetic pathway to limonoid. Open Nat. Prod. J..

[207] Huo C., Guo D., Shen L., Wang W., Zhang Q., Zhang M., Shi Q. (2010). One new limonoid from seeds of *Xylocarpus granatum*. Zhongcaoyao.

[208] Huo C.-H., Dong C., Shen L.-R., Yin B.-W., Sauriol F., Li L.-G., Zhang M.-L., Shi Q.-W., Kiyota H. (2010). Xylocarpanoids A and B, unique C28 skeleton limonoids from *Xylocarpus granatum*. Tetrahedron Lett..

[209] Kokpol U., Chavasiri W., Tip-pyang S., Veerachato G., Zhao F.L., Simpson J., Weavers R.T. (1996). A limonoid from *Xylocarpus granatum*. Phytochemistry.

[210] Li M.-Y., Wu J., Zhang S., Xiao Q., Li Q.-X. (2008). The absolute stereochemistry of protoxylogranatin A—A new protolimonoid from the seeds of Chinese mangrove *Xylocarpus granatum*. J. Asian Nat. Prod..

[211] Li M.-Y., Yang X.-B., Pan J.-Y., Feng G., Xiao Q., Sinkkonen J., Satyanandamurty T., Wu J. (2009). Granatumins A–G, limonoids from the seeds of a Krishna mangrove, *Xylocarpus granatum*. J. Nat. Prod..

[212] Ng A.S., Fallis A.G. (1979). Comment: 7α-Acetoxydihydronomilin and mexicanolide: Limonoids from *Xylocarpus granatum* (Koenig). Can. J. Chem..

[213] Okorie D.A., Taylor D.A.H. (1970). Limonoids from *Xylocarpus granatum* Koenig. J. Chem. Soc. C.

[214] Pan J.-Y., Chen S.-L., Li M.-Y., Li J., Yang M.-H., Wu J. (2010). Limonoids from the seeds of a Hainan mangrove, *Xylocarpus granatum*. J. Nat. Prod..

[215] Pudhom K., Sommit D., Nuclear P., Ngamrojanavanich N., Petsom A. (2009). Protoxylocarpins F-H, protolimonoids from seed kernels of *Xylocarpus granatum*. J. Nat. Prod..

[216] Shen L., Guo D., Yin B., Zhang Q., Zhao L., Huo C., Zhang M., Shi Q., Wang Y. (2009). Chemical constituents of *Xylocarpus granatum* Koenig. Zhongcaoyao.

[217] Waratchareeyakul W., Chantrapromma S., Fun H.-K., Razak I.A, Karalai C., Ponglimanont C. (2004). 7-Oxogedunin. Acta Crystallogr. Sect. E.

[218] Wu J., Li M.Y., Zhang S., Xiao Q., Li Q.X. (2007). Two new limonoids with a 3-*O*-β-tigloyl group from the seeds of the Chinese mangrove *Xylocarpus granatum*. Z. Naturforsch. Sect. B.

[219] Wu J., Zhang Z., Bruhn T., Xiao Q., Ding H., Bringmann G. (2008). Xylogranatins F–R: Antifeedants from the Chinese mangrove, *Xylocarpus granatum*, a new biogenetic pathway to triterpenoids. Chem. Eur. J..

[220] Yang X., Yang S., Li M., Pan J., Zheng Y., Wu J. (2010). Chemical constituents in seeds of Indian mangrove, *Xylocarpus granatum*. Zhongcaoyao.

[221] Yin S., Fan C.-Q., Wang X.-N., Lin L.-P., Ding J., Yue J.-M. (2006). Xylogranatins A–D: Novel tetranortriterpenoids with an unusual 9,10-*seco* scaffold from marine mangrove *Xylocarpus granatum*. Org. Lett..

[222] Yin S., Wang X.-N., Fan C.-Q., Lin L.-P., Ding J., Yue J.-M. (2007). Limonoids from the seeds of the marine mangrove *Xylocarpus granatum*. J. Nat. Prod..

[223] Yin B., Huo C., Shen L., Wang C., Zhao L., Wang Y., Shi Q. (2009). Protolimonoids from the seeds of *Xylocarpus granatum*. Biochem. Syst. Ecol..

[224] Cheng F., Zhou Y., Wu J. (2009). Chemical constituents of fruit of *Xylocarpus granatum*. Zhongyaocai.

[225] Cui J.X., Wu J., Deng Z.W., Proksch P., Lin W.H. (2007). Xylocarpins A–I, limonoids from the Chinese mangrove plant *Xylocarpus granatum*. J. Nat. Prod..

[226] Cui J., Ouyang J., Deng Z., Lin W. (2008). Structure elucidation of an unprecedented alkaloid and a new limonoid from *Xylocarpus granatum*. Magn. Res. Chem..

[227] Cui J., Deng Z., Xu M., Proksch P., Li Q., Liu W. (2009). Protolimonoids and limonoids from the Chinese mangrove plant *Xylocarpus granatum*. Helv. Chim. Acta.

[228] Wu J., Ding H.X., Li M.Y., Zhang S. (2007). Xylogranatin E, a new phragmalin with a rare oxygen bridge between C_1_ and C_29_, from the fruit of a Chinese mangrove *Xylocarpus granatum*. Z. Naturforsch. Sect. B.

[229] Wu J., Li M.Y., Xiao Z.H., Zhou Y.A. (2006). Butyrospermol fatty acid esters from the fruit of a Chinese mangrove *Xylocarpus granatum*. Z. Naturforsch. Sect. B.

[230] Wu J., Zhang S., Li M.Y., Zhou Y., Xiao Q. (2006). Xylogranatins A–D, new mexicanolides from the fruit of a Chinese mangrove *Xylocarpus granatum*. Chem. Pharm. Bull..

[231] Wu J., Zhihui X., Yang S., Zhang S., Xiao Q., Ma C., Ding H., Li Q. (2006). Complete assignments of ^1^H and ^13^C NMR data for two 3β,8β-epoxymexicanolides from the fruit of a Chinese mangrove *Xylocarpus granatum*. Magn. Res. Chem..

[232] Wu J.J. (2005). Two new mexicanolides from the fruit of the Chinese mangrove *Xylocarpus granatum*. Z. Naturforsch. Sect. B.

[233] Zhou Y., Cheng F., Wu J., Zou K. (2006). Polyhydroxylated phragmalins from the fruit of a Chinese mangrove, *Xylocarpus granatum*. J. Nat. Prod..

[234] Zhou Y., Wu J., Zou K. (2007). Xylogranatinin, a new pyrido[1,2-*a*]pyrazine alkaloid from the fruit of a Chinese mangrove *Xylocarpus granatum*. Chem. Nat. Comp..

[235] Shen L.-R., Guo D., Yu Y.-M., Yin B.-W., Zhao L., Shi Q.-W., Wang Y.-L., Huo C.-H. (2009). Chemical constituents of plants from the genus *Xylocarpus*. Chem. Biodiv..

[236] Phatthalung P.N., Chusri S., Voravuthikunchai S.P. (2012). Thai ethnomedicinal plants as resistant modifying agents for combating *Acinetobacter baumannii* infections. BMC Complement. Altern. Med..

[237] Alam M.Q., Sarder M., Awal M.A., Sikder M.M.H., Daulla K.A. (2006). Antibacterial activity of the crude ethanolic extract of *Xylocarpus granatum* stem barks. Bangladesh J. Vet. Med..

[238] Choudhury S., Sree A., Mukherjee S.C., Pattnaik P., Bapuji M. (2005). *In vitro* antibacterial activity of extracts of selected marine algae and mangroves against fish pathogens. Asian Fish. Sci..

[239] Misra S., Verma M., Mishra S., Srivastava S., Lakhsmi V., Misra-Bhattacharya S. (2011). Gedunin and photogedunin of *Xylocarpus granatum* possess antifilarial activity against human lymphatic filarial parasite *Brugia malayi* in experimental rodent host. Parasitol. Res..

[240] Lakhsmi V., Srivastava S., Mishran S.K., Srivastava M.N., Srivastava K., Puri S.K. (2012). Antimalarial activity in *Xylocarpus granatum* (Koen). Nat. Prod. Res..

[241] Lakhsmi V., Singh N., Shrivastva S., Mishra S.K., Dharmani P., Mishra V., Palit G. (2010). Gedunin and photogedunin of *Xylocarpus granatum* show significant anti-secretory effects and protect the gastric mucosa of peptic ulcer in rats. Phytomedicine.

[242] Uddin S.J., Nahar L., Shilpi J.A., Shoeb M., Borkowski T., Gibbons S., Middleton M., Byres M., Sarker S.D. (2007). Gedunin, a limonoid from *Xylocarpus granatum*, inhibits the growth of CaCo-2 colon cancer cell line *in vitro*. Phytother. Res..

[243] Uddin S.J., Grice I.D., Tiralongo E. (2011). Cytotoxic effects of Bangladeshi medicinal plant extracts. Evid. Based Complement. Altern. Med..

[244] Uddin S.J., Shilpi J.A., Alam S.M.S., Alamgir M., Rahman M.T., Sarker S.D. (2005). Antidiarrhoeal activity of the methanol extract of the barks of *Xylocarpus moluccensis* in castor oil- and magnesium sulphate-induced diarrhoea models in mice. J. Ethnopharmacol..

[245] Piyagaw, *Xylocarpus moluccensis*. http://stuartxchange.org/Piyagaw.html.

[246] Sarker S.D., Uddin S.J., Shilpi J.A., Rouf R., Ferdous M.E.M., Nahar L. (2007). Neuropharmacological properties of *Xylocarpus moluccensis*. Fitoterapia.

[247] Connolly J.D., MacLellan M., Okorie D.A., Taylor D.A.H. (1976). Limonoids from *Xylocarpus moluccensis* (Lam.) M. Roem. J. Chem. Soc. Perkin Trans. 1.

[248] Mulholland D.A., Taylor D.A.H. (1992). Limonoids from Australian members of the Meliaceae. Phytochemistry.

[249] Taylor D.A.H. (1983). Limonoid extractives from *Xylocarpus moluccensis*. Phytochemistry.

[250] Bercich M.G., Cambie R.C., Lal A.R., Sidwell D. (1998). Chemistry of Fijian plants. XIV. An unsaturated aryl keto acid from *Xylocarpus moluccensis*. Austr. J. Chem..

[251] Roy A.D., Kumar R., Gupta P., Khaliq T., Narender T., Aggarwal V., Roy R. (2006). Xyloccensin X and Y, two new limonoids from *Xylocarpus moluccensis*: NMR investigation in mixture. Magn. Res. Chem..

[252] Kubo I., Miura I., Nakanishi K. (1976). Structure of xylomollin, a secoiridoid hemiacetal acetal. J. Am. Chem. Soc..

[253] Li M.-Y., Yang S.-X., Pan J.-Y., Xiao Q., Satyanandamurty T., Wu J. (2009). Moluccensins A–G, phragmalins with a conjugated C-30 carbonyl group from a Krishna mangrove, *Xylocarpus moluccensis*. J. Nat. Prod..

[254] Li J., Li M.-Y., Feng G., Xiao Q., Sinkkonen J., Satyanandamurty T., Wu J. (2010). Limonoids from the seeds of a Godavari mangrove, *Xylocarpus moluccensis*. Phytochemistry.

[255] Li J., Li M.-Y., Satyanandamurty T., Wu J. (2011). Godavarin K: A new limonoid with an oxygen bridge between C(1) and C(29) from the Godavari mangrove *Xylocarpus moluccensis*. Helv. Chim. Acta.

[256] Li M.-Y., Zhang J., Feng G., Satyanandamurty T., Wu J. (2011). Khayasin and 2′*S*-methylbutanoylproceranolide: Promising candidate insecticides from the control of the coconut leaf beetle, *Brontispa longissima*. J. Pestic. Sci..

[257] Li J., Li M.-Y., Feng G., Zhang J., Karonen M., Sinkkonen J., Satyanandamurty T., Wu J. (2012). Moluccensins R–Y, limonoids from the seeds of a mangrove, *Xylocarpus moluccensis*. J. Nat. Prod..

[258] Pudhom K., Sommit D., Nuclear P., Ngamrojanavanich N., Petsom A. (2010). Moluccensins H–J, 30-ketophragmalin limonoids from *Xylocarpus moluccensis*. J. Nat. Prod..

[259] Ravangpai W., Sommit D., Teerawatananond T., Sinpranee N., Palaga T., Pengpreecha S., Muangsin N., Pudhom K. (2011). Limonoids from seeds of Thai *Xylocarpus moluccensis*. Bioorg. Med. Chem. Lett..

[260] Wu J., Yang S.-X., Li M.-Y., Feng G., Pan J.-Y., Xiao Q., Sinkkonen J., Satyanandamurty T. (2010). Limonoids and tirucallane derivatives from the seeds of a Krishna mangrove, *Xylocarpus moluccensis*. J. Nat. Prod..

[261] Zhang J., Yang S.-X., Yang X.-B., Li M.-Y., Feng G., Pan J.-Y., Satyanandamurty T., Wu J. (2010). Mexicanolides from the seeds of a Krishna mangrove, *Xylocarpus moluccensis*. Chem. Pharm. Bull..

[262] Gunawan S., Darmawan R., Nanda M., Setiawan A.D., Fansuri H. (2013). Proximate composition of *Xylocarpus moluccensis* seeds and their oils. Ind. Crops Prod..

[263] Li M., Wu J. (2010). Khayasin from *Xylocarpus moluccensis* for Control of Brontispa longissima larvae. Chinese Patent.

[264] Li M., Wu J. (2010). Application of Xylolimonoid A from *Xylocarpus moluccensis* as Insecticide for *Brontispa longissima*. Chinese Patent.

[265] Wisutsitthiwong C., Buranaruk C., Pudhom K., Palaga T. (2011). The plant limonoid 7-oxo-deacetoxygedunin inhibits RANKL-induced osteoclastogenesis by suppressing activation of the NF-κB and MAPK pathways. Biochem. Biophys. Res. Comm..

[266] Adzu B., Amos S., Amizan M.B., Gamaniel K. (2003). Evaluation of the antidiarrhoeal effects of *Zizyphus spina-christi* stem bark in rats. Acta Trop..

[267] Agbor G.A., Léopold T., Jeanne N.Y. (2004). The antidiarrhoeal activity of *Alchornea cordifolia* leaf extract. Phytother. Res..

[268] Atta A.H., Mouneir S.M. (2004). Antidiarrhoeal activity of some Egyptian medicinal plant extracts. J. Ethnopharmacol..

[269] Gabriel S.E., Davenport S.E., Steagall R.J., Vimal V., Carlson T., Rozhon E.J. (1999). A novel plant-derived inhibitor of cAMP-mediated fluid and chloride secretion. Am. J. Physiol. Gastrointest. Liver Physiol..

[270] Schuier M., Sies H., Illek B., Fischer H. (2005). Cocoa-related flavonoids inhibit CFTR-mediated chloride transport across T84 human colon epithelia. J. Nutr..

[271] Toda S., Shirataki Y. (2000). Inhibitory effects of sophoraflavanones B and H in *Sophora moorcroftiana* Beth ex Baker on copper-ion-induced protein oxidative modification of mice brain homogenate *in vitro*. Biol. Trace Element Res..

[272] Kaur T., Singh S., Dhawan V., Ganguly N.K. (1998). *Shigella dysenteriae* type 1 toxin induced lipid peroxidation in enterocytes isolated from rabbit ileum. Mol. Cell. Biochem..

[273] Rawal S., Majumdar S., Dhawan V., Vohra H. (2004). *Entamoeba histolytica* Gal/GalNAc lectin depletes antioxidant defences of target epithelial cells. Parasitology.

[274] Nieto N., López-Pedrosa J.M., Mesa M.D., Torres M.I., Fernández M.I., Ríos A., Suárez M.D., Gil A. (2000). Chronic diarrhea impairs intestinal antioxidant defense system in rats at weaning. Dig. Dis. Sci..

[275] Naito Y., Takagi T., Yoshikawa T. (2007). Neutrophil-dependent oxidative stress in ulcerative colitis. J. Clin. Biochem. Nutr..

[276] Tomassoni A.J., Simone K. (2001). Herbal medicines for children: An illusion of safety?. Curr. Opin. Pediatr..

[277] Farnsworth N.R., Chadwick D.J., Marsh J. (1994). Ethnopharmacology and Drug Development. Ethnobotany and the Search for New Drugs.

